# Changes in plastid biogenesis leading to the formation of albino regenerants in barley microspore culture

**DOI:** 10.1186/s12870-020-02755-z

**Published:** 2021-01-07

**Authors:** Monika Gajecka, Marek Marzec, Beata Chmielewska, Janusz Jelonek, Justyna Zbieszczyk, Iwona Szarejko

**Affiliations:** Faculty of Natural Sciences, Institute of Biology, Biotechnology and Environmental Protection, University of Silesia, Jagiellonska 28, Katowice, 40-032 Poland

**Keywords:** Albinism, Androgenesis, Chloroplast differentiation, Doubled haploids, *Hordeum vulgare*, Isolated microspore culture, Microspore embryogenesis, Plastid biogenesis, Plastid genome

## Abstract

**Background:**

Microspore embryogenesis is potentially the most effective method of obtaining doubled haploids (DH) which are utilized in breeding programs to accelerate production of new cultivars. However, the regeneration of albino plants significantly limits the exploitation of androgenesis for DH production in cereals. Despite many efforts, the precise mechanisms leading to development of albino regenerants have not yet been elucidated. The objective of this study was to reveal the genotype-dependent molecular differences in chloroplast differentiation that lead to the formation of green and albino regenerants in microspore culture of barley.

**Results:**

We performed a detailed analysis of plastid differentiation at successive stages of androgenesis in two barley cultivars, ‘Jersey’ and ‘Mercada’ that differed in their ability to produce green regenerants. We demonstrated the lack of transition from the NEP-dependent to PEP-dependent transcription in plastids of cv. ‘Mercada’ that produced mostly albino regenerants in microspore culture. The failed NEP-to-PEP transition was associated with the lack of activity of *Sig2* gene encoding a sigma factor necessary for transcription of plastid rRNA genes. A very low level of *16S* and *23S rRNA* transcripts and impaired plastid translation machinery resulted in the inhibition of photomorphogenesis in regenerating embryos and albino regenerants. Furthermore, the plastids present in differentiating ‘Mercada’ embryos contained a low number of plastome copies whose replication was not always completed. Contrary to ‘Mercada’, cv. ‘Jersey’ that produced 90% green regenerants, showed the high activity of PEP polymerase, the highly increased expression of *Sig2*, plastid *rRNAs* and *tRNA*^*Glu*^, which indicated the NEP inhibition. The increased expression of *GLKs* genes encoding transcription factors required for induction of photomorphogenesis was also observed in ‘Jersey’ regenerants.

**Conclusions:**

Proplastids present in microspore-derived embryos of albino-producing genotypes did not pass the early checkpoints of their development that are required for induction of further light-dependent differentiation of chloroplasts. The failed activation of plastid-encoded RNA polymerase during differentiation of embryos was associated with the genotype-dependent inability to regenerate green plants in barley microspore culture. The better understanding of molecular mechanisms underlying formation of albino regenerants may be helpful in overcoming the problem of albinism in cereal androgenesis.

## Background

Isolated microspore culture *via* microspore embryogenesis is the most effective of haploid/doubled haploid (DH) production methods that are used in plant breeding programmes to shorten the time required for development of new varieties [1–3]. Microspores, which originate from reduction division of pollen mother cells, develop in vivo into pollen grains [4, 5]. By the exposition to stress factors such as starvation or temperature regime, microspores can be induced to enter the path of embryogenesis [6]. Stress treatment or pre-treatment reprograms the gametophytic pathway of microspore development into the sporophytic one (androgenesis), which results in induction of embryo formation and regeneration of androgenic plants [7, 8]. Despite the available optimized protocols for induction of microspore embryogenesis, the process remains highly genotype-dependent. Moreover, even if a sufficient number of regenerants is produced in culture, the effectiveness of androgenesis in cereals is vastly limited by regeneration of albino plants, which reduces the utilisation of androgenesis in breeding programs. Albinism occurring in androgenic cultures is a phenomenon that is distinctive for cereals and grasses, and occurs in a wide frequency range in nearly all agronomically important monocot species, including wheat [9, 10], rice [11], barley [12, 13] and triticale [14].

Regeneration of green plants is possible when proplastids enclosed in microspores, that in vivo develop into amyloplasts [15], differentiate into chloroplasts during in vitro culture. Yet, not all proplastids present in cultured microspores develop into chloroplasts. Some of them follow the same developmental pathway as proplastids during in vivo pollen development and differentiate into amyloplasts, some do not progress into plastids and degenerate. In such cases, microspore-derived embryos do not contain plastids capable to carry photosysnthesis and the regenerated albino plants are unable to grow outside the in vitro cultures [16]. Albinism is a highly genotype-dependent process and the frequency of regeneration of albino plants among cultivars varies between 1 to 100%, which shows that some genotypes are more prone to the regeneration of albino plants [17]. Our previous study that focused on biogenesis and differentiation of plastids during development of pollen grains in vivo indicated that the genotype-dependent regeneration of albino plants in barley is determined by the time of activation of starch-synthesis apparatus related to proplastid-to-amyloplast transition during microgametogenesis [18]. Furthermore, as a primary cause of albino plants formation in vitro, deletions in the plastid genome were indicated in barley [19–21], triticale [22], wheat [19, 22, 23] and rice [24, 25]. However, another study showed many differences in expression level of plastid-encoded genes between albino and green regenerants but no changes in the plastid genome [26]. Attempts have been also made to identify QTLs associated with the frequency of green plant regeneration in barley [27, 28] and triticale [29]. Yet, the detailed mechanism leading to the detention of chloroplast differentiation has not been revealed.

Chloroplasts contain their own genome (plastome, ptDNA) that is represented by many copies (10–500 copies per one plastid) included in the stroma [30, 31]. Approximately 140 genes are located in the plastome that encode subunits of plastid-encoded polymerase (PEP), proteins of ribosome subunits, plastid rRNAs and tRNAs, proteins facilitating import of the nuclear-encoded proteins and proteins of photosynthetic apparatus [32, 33]. Nevertheless, ptDNA contains only small amount of information that is required for development of functional organelle and most of the proteins are encoded in the nuclear genome. Thus, the differentiation of plastids requires the coordinated expression of plastid-encoded and several thousands of nuclear-encoded genes [34], and depends on the nucleus-to-plastid (anterograde signalling) and plastid-to-nucleus (retrograde signalling) communication. This communication is necessary for all processes regarding the chloroplast formation, including plastome integrity, transcription, translation and assembly of photosynthetic complexes [30, 35, 36]. Anterograde communication involves import of nuclear-encoded proteins synthesised in cytosol that is carried out mainly by TIC/TOC complex [37, 38]. Any knockout mutation resulting in a lack of proteins that are assembled in the import complexes blocks chloroplast differentiation and leads to the albino phenotype [37]. The communication of nucleus-to-plastid is also required for transcription occurring in plastids that is performed by two RNA polymerases: NEP (nuclear-encoded polymerase) and PEP (plastid-encoded polymerase). NEP and PEP recognise specific promoters and transcribe partially coinciding and specific set of genes [39–41]. During early stage of plastid biogenesis NEP plays a crucial role in transcription of plastid-localised genes related to plastid biogenesis including transcription and translation. NEP is a single-subunit phage-type RNA polymerase encoded by *RpoTp* gene. Dicots harbour additional NEP polymerase encoded by *RpoTmp* gene that transcribes genes in plastids and mitochondria [42]. During progression of proplastid-to-chloroplast differentiation the main role in plastid genes transcription is acquired by bacterial-type PEP that consists of five subunits: two α and β, β’, β” encoded respectively by plastid *rpoA* and *rpoB*, *rpoC1*, *rpoC2* genes assembled in separate operons [43]. PEP for its action requires sigma factors that are encoded in the nuclear genome by *Sig1*-*Sig6* genes and enable recognition of specific promoters and initiation of transcription by the PEP holoenzyme. Upon illumination, the PEP holoenzyme is reconstructed by the addition of PEP-associated proteins (PAPs) that are required for the formation and/or stabilization of the PEP complex and regulation of transcription. Lack of any of PAPs leads to the deficiency of PEP-dependent transcription at the early stage of chloroplast development which results in albino phenotype of mutants [43].

Among sigma factors, SIG2 and SIG6 are required in early plastid biogenesis and are positively regulated in response to light by phytochromes A and B [44–46]. SIG2-dependent transcription includes *tRNA*^*Glu*^ that plays a dual role: as a component in translation during chloroplast differentiation and as a substrate in synthesis of 5-aminolevulinic acid (ALA) which is a precursor of chlorophyll. ALA synthesis correlates with control of nuclear gene expression mediated by retrograde signalling [47, 48]. At early phase of plastid biogenesis, the SIG2 imported to plastid forms a complex with the PEP and initiates the transcription of *tRNA*^*Glu*^*.* The high level of *tRNA*^*Glu*^ transcripts inhibits the activity of NEP by binding to it [49, 50]. PEP provides a high level of transcripts of plastid-localised genes which is required for effective assembling of chloroplasts [51]. Plastid mRNAs are translated by the plastid translation machinery that involves 70S ribosome built of proteins encoded by nuclear and plastid genes and plastid-encoded rRNAs. All tRNAs required for translation occurring in plastids are encoded in the plastome. Among factors involved in initiation, elongation and termination of translation only one, IF1 (Initiation factor1) is encoded by a gene (*infA*) localised in the plastome [52, 53].

Proper transcription and translation occurring in proplastid as well as integral plastome are the first checkpoints during plastid and are required to induce further light-dependent maturation of chloroplast including assembly of the PEP holoenzyme which must be finished before photomorphogenesis can be performed [54, 55]. Light-activated phytochromes bind transcription factors PIFs (Phytochrome-Interacting Factors) which are repressors of photomorphogenesis and suppress differentiation of chloroplast in darkness [31, 56]. PIFs degradation initiates expression of transcription factors reminding under PIFs control including GLKs (Gloden-like) and HY5 (Long Hypocotyl5) whose activation induces expression of photosynthesis-associated nuclear genes (PhANGs) and other nuclear-encoded genes related to chlorophyll synthesis [57–59]. Overexpression of GLKs induced chloroplast development and greening of Arabidopsis roots [60] and rice callus tissue [61]. Deprivation of light induces skotomorphogenesis and development of etioplasts that contain protochlorophyllide and prolamellar body [62]. Many photosynthesis-related proteins were identified in etioplasts including subunits of ATP synthase, RubisCo and cytochrome *b6f* complexes [63, 64]. Under exposure of etiolated tissues to light, the development of chloroplasts and greening occurs which indicates that the prolamellar body is the precursor of thylakoids [65]. Alterations in plastid biogenesis, including plastid transcription, translation and signalling, frequently lead to the albino phenotype [34], therefore the role of plastid to chloroplast differentiation during androgenic culture seems to be crucial in transition of microspore plastids to fully assembled and functional chloroplasts.

In our previous studies on differentiation of plastids during microsporogenesis in vivo we found that barley cultivars producing mostly albino regenerants in androgenesis showed early activation of starch synthesis genes, differentiation of proplastids into amyloplasts and degradation of plastomes during microspore development [18]. Using several barley genotypes that presented different ability to regenerate green plants we confirmed the correlation between plastid differentiation prior to culture and albino regeneration in culture. However, we have not analysed the molecular mechanisms leading to regeneration of green and albino regenerants from microspore-derived embryos during in vitro culture. Here we present, for the first time, a detailed analysis of plastid differentiation at successive stages of androgenic embryo formation and plant regeneration in two barley cultivars that differ in their ability to produce green regenerants in isolated microspore culture. Based on the analysis of expression profiles of genes involved in transcription and translation occurring in plastids and genes related to chloroplast differentiation, together with determining the plastome copy number and analysis of plastid morphology and ultrastructure, we indicated the mechanisms that underlie the alterations in chloroplast development leading to the formation of albino plants. We revealed the molecular processes responsible for the failed activation of plastome transcription in the albino-producing genotype and discuss the mechanisms of chloroplast formation that differed in both genotypes.

## Results

### Androgenic potential of ‘Jersey’ and ‘Mercada’ cultivars

**Fig. 1:**
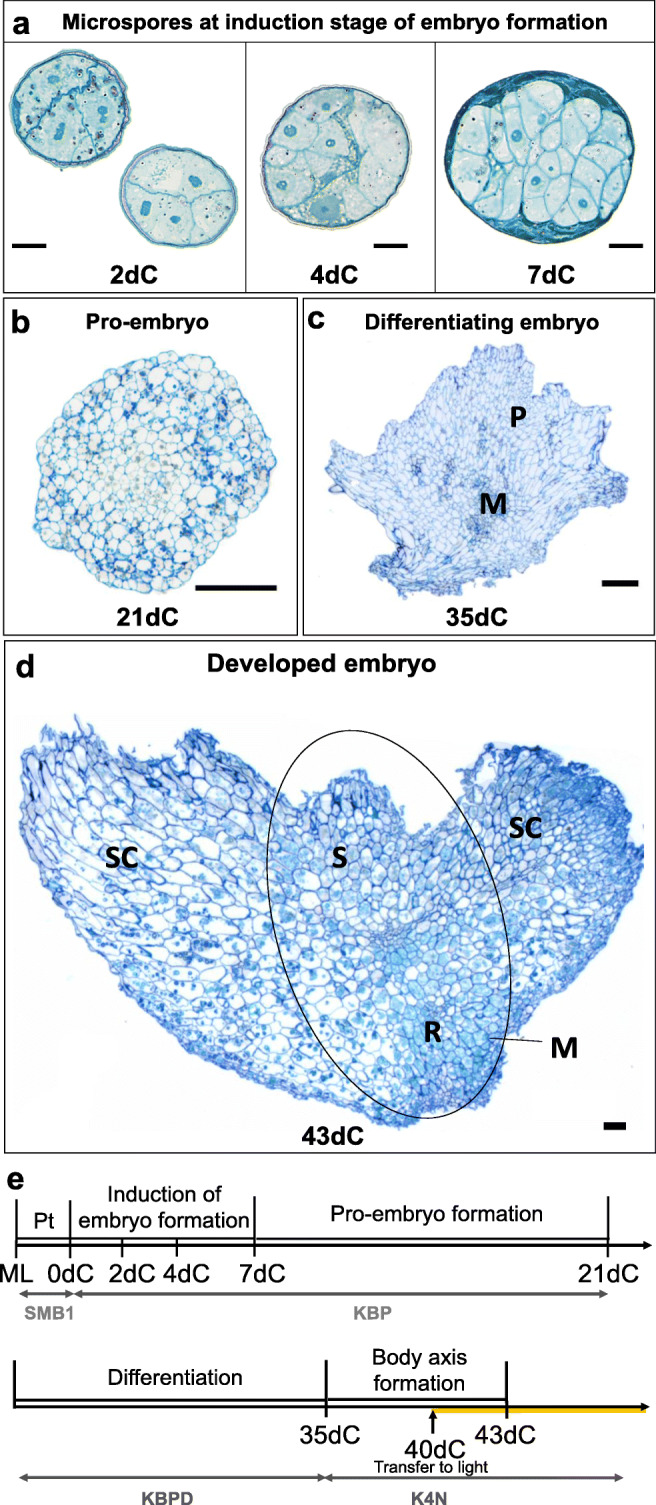
Development of androgenic embryos in isolated microspore culture of barley cvs. ‘Jersey’ and ‘Mercada’. **a-d** Tangential sections of androgenic embryos including: induction phase of embryo formation (**a**), a formed pro-embryo on 21dC (**b**), differentiating embryo on 35dC (**c**) and fully developed embryo on 43dC with formed body axis (**d**). **e** The overview of androgenic embryo development during isolated microspore culture. The culture is initiated from mid-to-late microspores (ML) that are pre-treated for two days in SMB1 medium, followed by a 3-week culture in KBP induction medium in the dark. Next, developed pro-embryos are transferred onto KBPD differentiation medium for two weeks for differentiation. On 35dC the differentiating embryos are transferred onto K4NB regeneration medium to form embryo body axis, first in the dark and since 40dC in light. dC –day of culture, M – meristematic zone, ML – mid-to-late microspore, P – parenchyma cells, Pt – pre-treatment, R - root apical meristem, S – shoot apical meristem, SC – scutellum. Scale bars: 20 μm in a; 100 μm in b-d

**Fig. 2:**
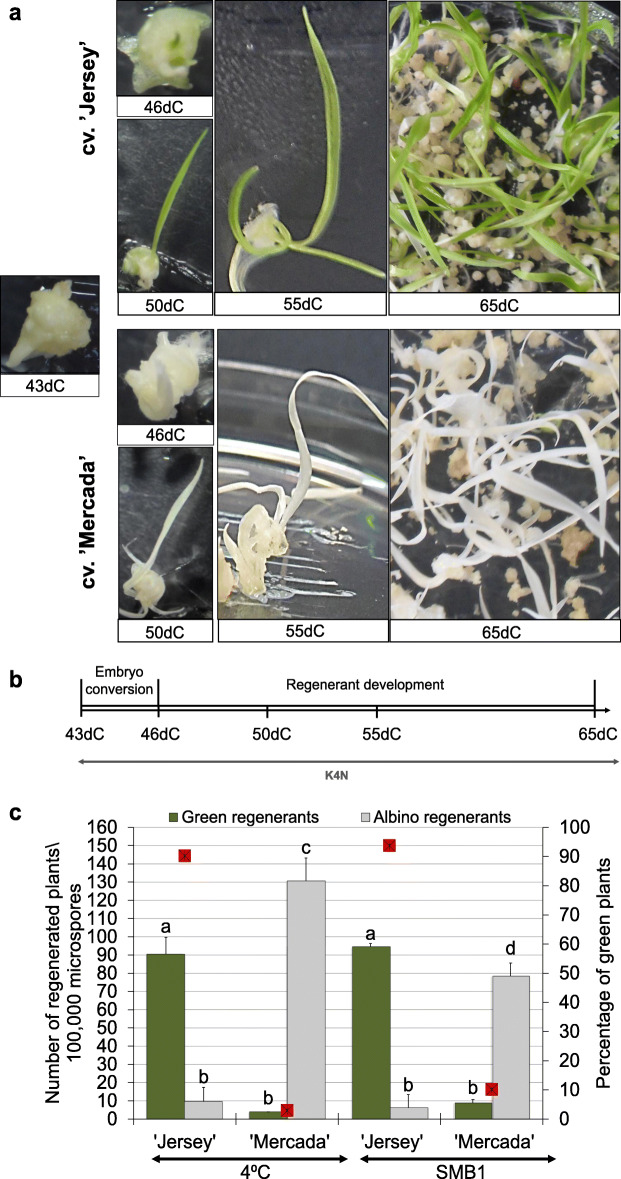
The types of regenerants developed in isolated microspore culture of ‘Mercada’ and ‘Jersey’ cultivars. **a** Subsequent phases of plant regeneration in microspore culture of cv. ‘Jersey’ and of cv. ‘Mercada’. **b** The overview of embryo conversion and regenerant development starting from developed androgenic embryo on 43dC. **c** The average number of regenerants obtained in isolated microspore culture initiated after cold or SMB1 pre-treatment. Given values present mean of *n* ≥ 3 with SD. Different letters indicate a significant difference between cultivars and pre-treatments according to Tukey’s HSD test (*P* < 0.05). dC – day of culture, GP – green plants. A red square indicates a percentage of green regenerants

Plant regeneration in isolated microspore culture of cvs. ‘Jersey’ and ‘Mercada’ occurred via process of microspore embryogenesis in which several phases could be distinguished. The first stage after pretreatment - induction of sporophytic development, involved cell divisions within the exine wall that led to the formation of a multicellular structure on the 7th day of culture (7dC, Fig. 1a). Rapture of the exine wall and further cell divisions led to the formation of structures that could be observed on the 21st day of culture (21dC) as globular pro-embryos (Fig. 1b). At this stage, the developed pro-embryos were transferred onto differentiation medium for 2 weeks (Fig. 1e). On the 35th day of culture (35dC), the meristematic zone could be recognised in differentiating embryos (Fig. 1c). Then, the embryos were transferred onto regeneration medium, for the first 5 days in dark and next in light (Fig. 1e). Already after 8 days on regeneration medium, on the 43rd day of culture (43dC), the embryo body axis was detectable (Fig. 1d). The fully developed androgenic embryos, ready to convert into plants were visible on 46th day of in vitro culture (46dC, Fig. 2a). The further stages of plant regeneration presented in Fig. 2a, b refer to the time points used in this study for molecular analysis. It should be underlined that microspore-derived embryos of both cultivars reached the same stage of development at the same time.

Both cultivars selected for the study, ‘Jersey’ and ‘Mercada’, exhibited a high regeneration potential in isolated microspore culture and produced over 100 plants per 100,000 plated microspores. Along with the high overall number of regenerants, ‘Jersey’ and ‘Mercada’ differed in the frequency of regeneration of green plants. ‘Jersey’ regenerated 90% green plants whereas ‘Mercada’ produced only 5–10% green regenerants and the majority of regenerants were albino, irrespectively of the stress treatment applied for inducing of androgenesis (Fig. 2c). A slight increase in the contribution of green plants among regenerants was observed in ‘Mercada’ culture after SMB1 pre-treatment, but it was accompanied with reduction of the overall regeneration ability, compared to the cold pre-treatment (Fig. 2c). These results suggest that the modification of inductive treatment did not overcome the genotype-dependent formation of albino regenerants in barley androgenesis.

### Plastid biogenesis and development during microspore-derived embryo formation and differentiation

**Fig. 3:**
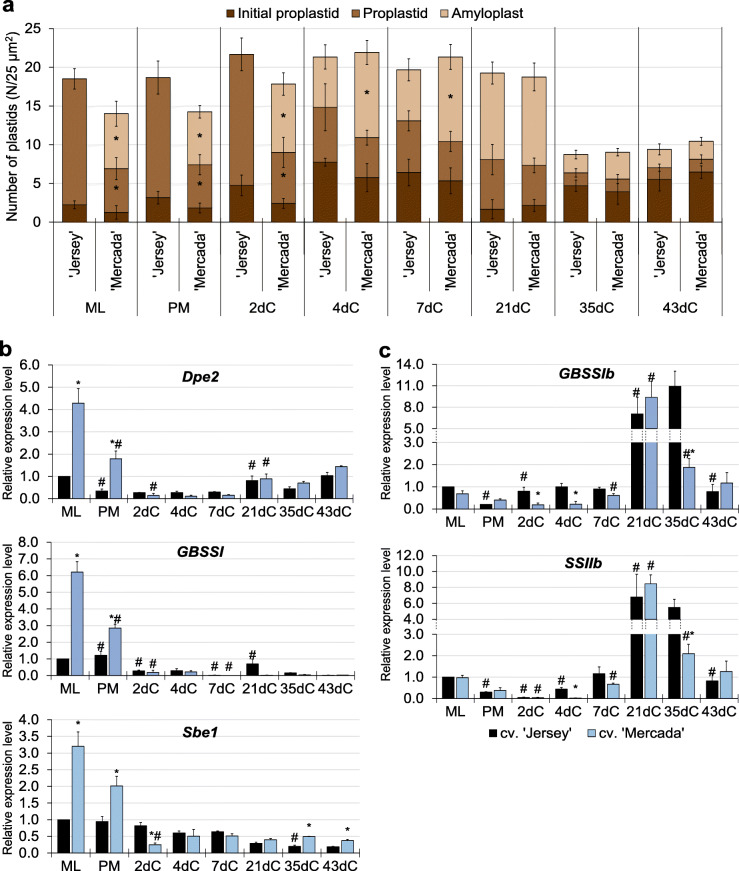
Plastids density and expression of starch synthesis genes during embryo formation in ‘Jersey’ and ‘Mercada’ cultures. **a** Types and density of plastids present in microspores and microspore-derived embryos at successive stages of culture. Initial proplastid was characterised by a low electron density and the presence of single invaginations from the inner membrane, the differentiating proplastid by much higher electron density and single invaginations from the inner membrane, amyloplast was filled with starch grains. Values present mean of *n* ≥ 3 with SD. **b**, **c** The relative expression level of genes related to reserve starch (**b**) and assimilatory starch (**c**) synthesis in ‘Jersey’ and ‘Mercada’ cultures. Graphs show mean values of *n ≥* 3 with SEM. Relative expression level was normalised to ML microspores of cv. ‘Jersey’. An asterisk presents a value significantly different between cultivars at a certain day of culture. A hash indicates a value significantly different from the preceding day of culture within cultivar (Tukey’s test, *P* < 0.05). ML – mid-to-late microspore, PM – pre-treated microspores, dC – day of culture

At the stage of culture initiation, cvs. ‘Mercada’ and ‘Jersey’ showed differences in the types of plastids presented in the mid-to-late (ML) microspores utilised to initiate in vitro culture. TEM observation allowed to distinguish different types of plastids harboured by microspores and to estimate their numbers. The number of plastids was assessed per 25 μm^2^ of microspore cytoplasm and then per 25 μm^2^ of embryo cell cytoplasm in each cultivar. Cv. ‘Mercada’, that produced mostly albino plants in androgenesis, contained 50% amyloplasts that were filled with starch grains and 50% proplastids, whereas cv. ‘Jersey’ contained only initial and differentiating proplastids. The initial (undifferentiated) proplastids showed the single invaginations from the inner membrane and a low electron density, while the electron density of the differentiating proplastids was much higher [18]. After pre-treatment, till 2dC, no changes occurred in the number and types of plastids presented in both cultivars. On 4dC, the proplastid differentiation into amyloplasts was observed in cv. ‘Jersey’ and the increase in the number of amyloplasts in cv. ‘Mercada’. At the end of pro-embryo formation, on 21dC, the number and types of plastids were similar in both cultivars, with ca. 60% of amyloplasts (Fig. 3a). Interestingly, at the end of differentiation phase, on 35dC, the number of plastids rapidly decreased in both cultivars, and initial proplastids represented 50% of observed plastids. The similar number and types of plastids were observed in apical domain of fully developed androgenic embryos of both cultivars (43dC) which implies that androgenic embryos contain the same categories of plastids, irrespectively of the types of produced regenerants.

**Fig. 4:**
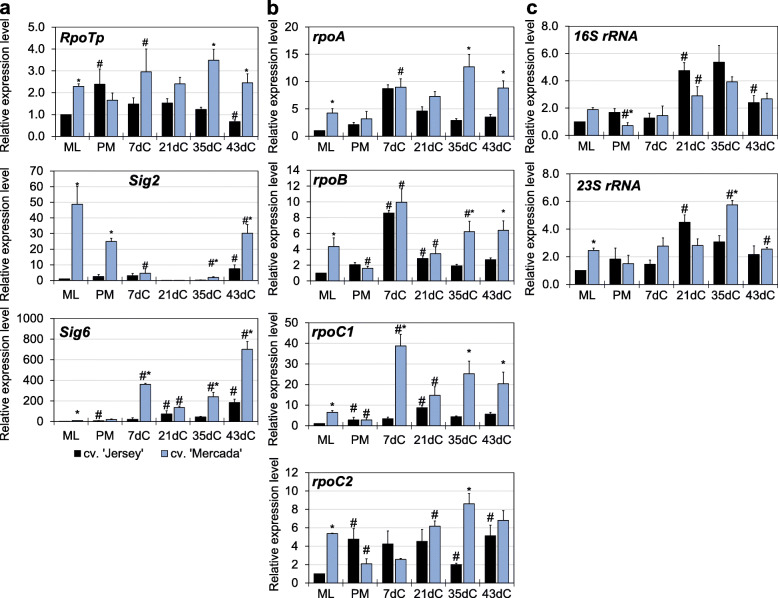
The expression profiles of plastid biogenesis genes during embryo formation in ‘Jersey’ and ‘Mercada’ cultures. **a**, **b** Relative expression level of nuclear-encoded (**a**) and plastid-encoded (**b**) genes related to plastid transcription: NEP (*RpoTp*), sigma factors (*Sig2*, *Sig6*) and PEP (*rpoA*, *rpoB*, *rpoC1* and *rpoC2*). **c** Relative expression level of plastid-encoded *16S* and *23S rRNA* genes. Graphs show mean values of *n ≥* 3 with SEM. The relative expression level was normalised to ML microspores of cv. ‘Jersey’. An asterisk presents a value significantly different between cultivars at a certain day of culture. A hash indicates a value significantly different from the preceding day of culture within cultivar (Tukey’s test, *P* < 0.05). ML – mid-to-late microspore, PM – pre-treated microspores, dC – day of culture

Taking into account the presence of amyloplast in microspore-derived embryos of both cultivars, we analysed the expression of genes encoding isoforms of enzymes involved in reserve and assimilatory starch synthesis (Additional file 1: Table S1). At the stage of culture initiation, in ML microspores the expression level of genes *Dpe2*, *GBSSI* and *Sbe1* involved in reserve starch synthesis was significantly higher in cv. ‘Mercada’, which was in accordance with the presence of amyloplasts observed exclusively in microspores of this cultivar (Fig. 3b). During isolated microspore culture, the expression of these genes decreased in both cultivars, which indicates the inhibition of reserve starch synthesis after stress treatment and induction of androgenesis. Contrary, the expression of *GBSSIb* and *SSIIb* genes encoding enzymes of assimilatory starch synthesis increased gradually after pre-treatment in both cultivars (Fig. 3c). In cv. ‘Jersey’ the increase in expression of *GBSSIb* and *SSIIb* genes was observed already on 2dC and 4dC, respectively, which explains the presence of starch-accumulating plastids observed in this cultivar on 4dC. The highest expression level of *GBSSIb* and *SSIIb* genes was observed on 21dC in developed pro-embryos of both cultivars. Interestingly, during embryo induction and pro-embryo development on 7dC and 21dC, no significant differences were observed between tested cultivars in the expression level of most genes related to plastid biogenesis that are involved in transcription and translation occurring in plastids. It should be noted that expression of these genes was higher in the cv. ‘Mercada’ compared to ‘Jersey’ at the stage of culture initiation (Fig. 4; Additional file 1: Fig. S1) and expression of most of the genes involved in transcription including NEP (*RpoTp* gene), PEP subunits (encoded by genes *rpoA*, *rpoB*, *rpoC1*, *rpoC2*) was again higher in cv. ‘Mercada’ at the end of embryo differentiation and body axis formation (35dC and 43dC; Fig. 4). Such differences were not observed for the majority of genes involved in translation that encode plastid rRNA (Fig. 4c) and proteins of ribosome subunits (Additional file 1: Fig. S1).

Taking into account these data, we concluded that green and albino producing cultivars did not show any substantial differences in plastid types and numbers during androgenic embryo induction and differentiation stage, however the developed embryos on 35dC and 43dC differed at the expression level of genes related to plastid biogenesis.

### The fluctuation of plastome copy numbers during isolated microspore culture

**Fig. 5:**
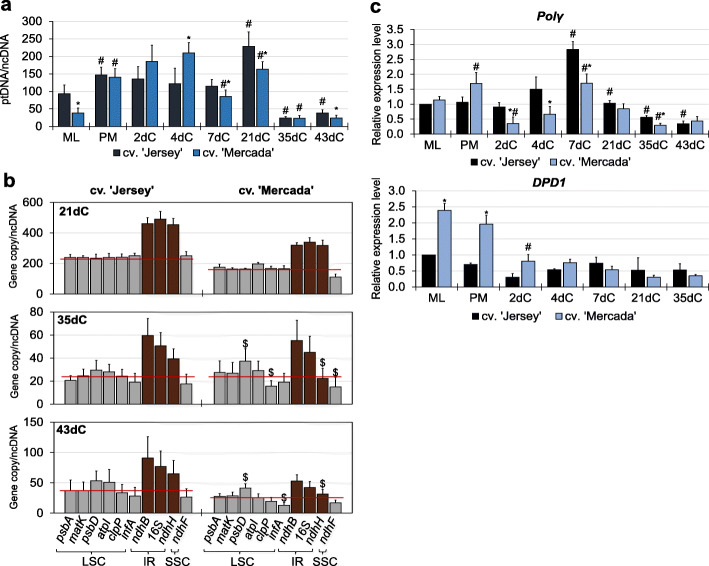
Plastid DNA content during isolated microspore culture of ‘Jersey’ and ‘Mercada’ cultivars. **a** Average plastome copy number in relation to nuclear genome in subsequent days of isolated microspore culture. **b** The individual copy number of genes localised in the plastid genome in subsequent days of isolated microspore culture of ‘Jersey’ and ‘Mercada’ cultivars. **c** The relative expression profile of *Polγ* (*Organellar DNA polymeraseI*) gene and *DPD1* gene encoding Mg^2+^ -dependent organelle exonuclease. Graphs show mean values of *n ≥* 3 with SD in **a** and **b** or SEM in **c**. Relative expression level was normalised to ML microspores of cv. ‘Jersey’. An asterisk presents a value significantly different between cultivars at a certain day of culture. A hash indicates a value significantly different from the preceding day of culture within cultivar (Tukey’s test, *P* < 0.05). A $ indicates a value significantly different from the calculated average plastome copy number at a particular day of culture within cultivar (Student’s t-test, *P* < 0.05). Red lines show the average copy numbers calculated from individual copies of presented genes. LSC – long single copy, IR – inverted repeat, SSC – short single copy, ML – mid-to-late microspore, PM – pre-treated microspores, dC – day of culture

Together with the plastid number, we assessed the number of plastid genomes during isolated microspore culture using qPCR. During culture initiation and embryo formation, the plastome copy number, which in ML microspores was twice as high in cv. ‘Jersey’ than ‘Mercada’, increased significantly in both cultivars and reached the highest value on 21dC (Fig. 5a). During differentiation of androgenic pro-embryos, between 21dC and 35dC, the rapid decrease (9 and 7 times in cv. ‘Jersey’ and ‘Mercada’, respectively) in plastome copy number was detected, parallel to the observed reduction in the number of plastids. However, while the number of plastids in the developed androgenic embryos on 43dC remained similar in both cultivars, the plastome copy number in plastids of cv. ‘Mercada’ was twice lower than in cv. ‘Jersey’ (Fig. 5a). Additionally, since the 35dC and the decline in the plastome copy number, the significant deviation of the copies of individual plastid genes was observed in cv. ‘Mercada’ (Fig. 5b).

The increase of the plastome copy number after pre-treatment indicated the induction of plastome replication process, therefore we analysed the expression profile of *Polγ* gene encoding the organellar DNA polymerase called also PolIA [66]. Expression of *Polγ* gene was similar in ML microspores of both cultivars (Fig. 5c). During embryo induction phase, from 2dC to 7dC, the *Polγ* expression level in cv. ‘Mercada’ was much lower than in cv. ‘Jersey’ but equalized at the end of pro-embryo formation (21 dC, Fig. 5c). Expression of *Polγ* gene profiled similarly in both cultivars during embryo differentiation and body axis formation. The increased number of plastome copies observed since 46dC (Additional file 1: Fig. S3) suggests that plastome replication was still active in both cultivars.

Since the culture is induced from immature pollen, we analysed the expression level of *DPD1* gene encoding organelle exonuclease that acts during pollen development in vivo and degrades organellar DNA during plastid differentiation. At the stage of culture initiation, the expression level of *DPD1* gene was 2–2.5 times higher in ML and pre-treated microspores of cv. ‘Mercada’ than cv. ‘Jersey’ and decreased during culture initiation (Fig. 5c). *DPD1* gene was active in cv. ‘Mercada’ already in microspores at the early stage of development, before culture initiation (Additional file 1: Fig. S2). This suggests an earlier activation of plastome degradation in cv. ‘Mercada’ during microspore development preceding culture initiation.

### Differences in plastid biogenesis between cvs. ‘Jersey’ and ‘Mercada’ during regeneration of microspore-derived plants

Despite the similar average number of plastome copies in both cultivars at the end of body axis formation phase (43dC), the deviation in the copy number of particular plastid genes could possibly affect the further plastid development, therefore we continued the analysis of gene expression involved in plastid biogenesis (Additional file 1: Table S1) during regeneration of androgenic plants (46dC, 50dC and 55dC).

The average plastome copy number in cvs. ‘Jersey’ and ‘Mercada’ increased in converting embryos and regenerated plants compared to 43dC, which indicated the replication of plastid genomes, correlated with the increased expression of *Polγ* gene in cv. ‘Jersey’ (Additional file 1: Fig. S3a,c). However, as we observed previously in. cv. ‘Mercada’ during embryo development, also during embryo conversion and regenerant development the copy number of particular genes did not represent the expected value regarding their localisation in the plastid genomes (Additional file 1: Fig. S3b).

**Fig. 6:**
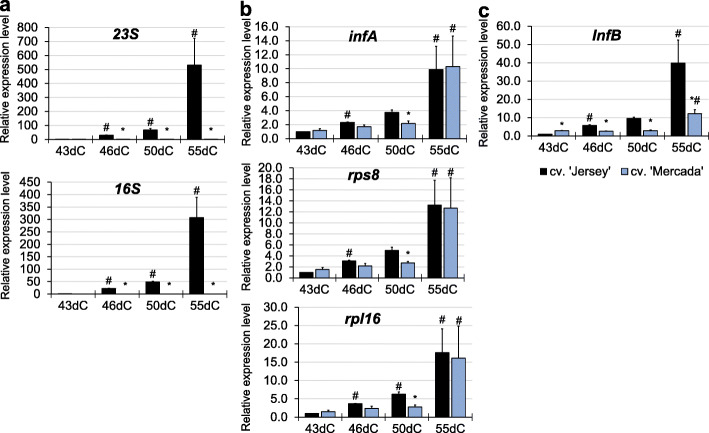
The expression profiles of translation-related genes during regeneration of cvs. ‘Jersey’ and ‘Mercada’ plants. **a** The relative expression level of plastid *16S* and *23S rRNA* genes. **b, c** The relative expression level of plastid- (**b**) and nuclear- (**c**) localised genes related to translation occurring in plastids, encoding: components of small ribosomal subunit (*rps8*), large ribosomal subunit (*rpl16*) and translation initiation factors (*infA*, *InfB*). Graphs show mean values of *n ≥* 3 with SEM for relative expression level normalised to 43dC of cv. ‘Jersey’. An asterisk presents a value significantly different between cultivars at a certain day of culture. A hash indicates a value significantly different from the preceding day of culture within cultivar (Tukey’s test, *P* < 0.05). dC – day of culture

What was the most striking, during regeneration of cv. ‘Jersey’ plants, the expression of *16S* and *23S* genes encoding plastid rRNA increased immensely since 46dC (20 to 30-fold) to reach 300–500 times higher level on 55dC. In contrast to ‘Jersey’, during regeneration of cv. ‘Mercada’ plants, we did not observe any significant increase in the level of plastid-encoded rRNA transcripts between 46dC and 55dC (Fig. 6a). The expression of *16S* and *23S* genes in ‘Mercada’ albino regenerants on 55dC remained at the same level as in 43-day old embryos, which had only the visible body axis. The expression of other tested genes related to translation: *rps8*, *rpl16* encoding proteins of small and large subunits of ribosome and *infA*, *InfB* encoding translation initiation factors increased in both cultivars during regeneration (Fig. 6b, c). Nonetheless, this growth was observed in cv. ‘Jersey’ already on 46dC, while in cv. ‘Mercada’ on 55dC in developed regenerants. On 55dC the level of expression of translation-related genes, except for *InfB,* was similar in both cultivars.

We showed that the vast increase in plastid rRNA transcript level was correlated with the regeneration of green plants during conversion of androgenic embryos. As a high level of expression of plastid *rRNA* genes is provided only by transcription carried by PEP (Plastid-encoded RNA polymerase), we analysed expression of other genes related to the process of transcription occurring in plastids. They were genes encoding proteins such as NEP (Nuclear-encoded RNA polymerase), subunits of PEP, SIG2 (sigma factor2 involved in transcription of plastid tRNAs) and *trnE* (*tRNA*^*Glu*^), all known to be involved in the switch between NEP- and PEP-dependent transcription [47, 49].

**Fig. 7:**
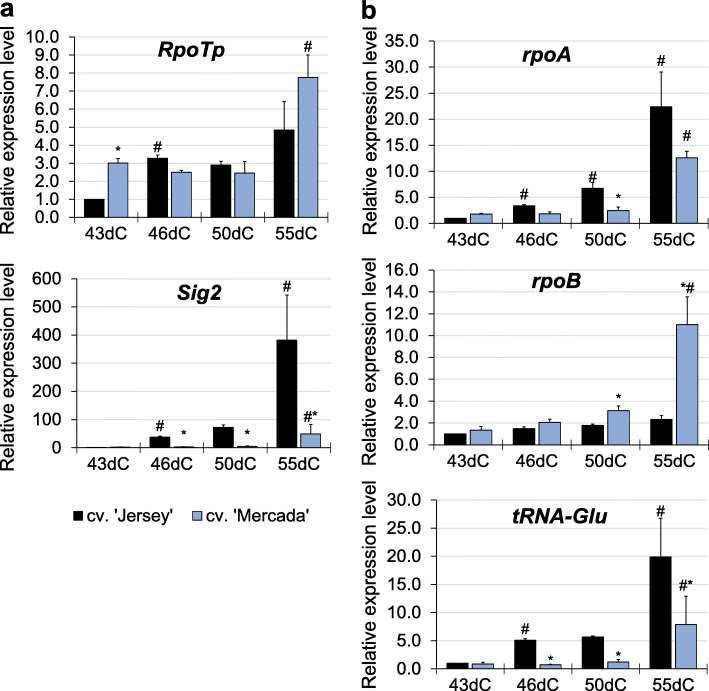
The expression profiles of transcription-related genes during regeneration of cvs. ‘Jersey’ and ‘Mercada’ plants. **a**, **b** The relative expression level of plastid- (**a**) and nuclear- (**b**) localised genes related to transcription occurring in plastids, encoding: NEP (nuclear-encoded polymerase; *RpoTp* gene), sigma factor (*Sig2*), α (*rpoA*) and β (*rpoB*) subunits of PEP (plastid-encoded polymerase) and *tRNA-E* (*tRNA*^*Glu*^) involved in communication between nucleus and plastid. Graphs show mean values of *n ≥* 3 with SEM for relative expression level normalised to 43dC of cv. ‘Jersey’. An asterisk presents a value significantly different between cultivars at a certain day of culture. A hash indicates a value significantly different from the preceding day of culture within cultivar (Tukey’s test, *P* < 0.05). dC – day of culture

In androgenic embryos of cv. ‘Mercada’ the expression level of *RpoTp* gene encoding NEP was three times higher than in cv. ‘Jersey’ on 43dC. In the further stages of regeneration the expression of this gene was similar in both cultivars, with the highest level in cv. ‘Mercada’ on 55dC (Fig. 7a). Two plastid-localised genes *rpoA* and *rpoB* encoding subunits α and β of PEP showed diverged profiles of expression between cvs. ‘Jersey’ and ‘Mercada’. In cv. ‘Jersey’, expression of *rpoA* increased gradually during embryo conversion and reached the highest level on 55dC, while in ‘Mercada’ the increase in *rpoA* transcript level could be observed only on 55dC (Fig. 7b). It should be noted that the plastid-localised *rpoA* gene is transcribed preferentially in greening leaves by the PEP itself [42, 67], so a low level of its transcription indicated a low activity of PEP in regenerating ‘Mercada’ embryos. Contrary to the *rpoA,* the *rpoB* gene encoding the β subunit of PEP, is preferentially transcribed by the NEP [39]. The transcription level of *rpoB* remained low and unchanged in ‘Jersey’ throughout the whole regeneration phase, while in ‘Mercada’ it increased significantly on 55dC compared to 43dC (Fig. 7b).

The high level of *rpoB* transcripts in ‘Mercada’ plants and differences in expression profiles of *RpoTp* and *rpoA* genes between both cultivars suggested that NEP was constantly active in plastids of regenerating ‘Mercada’ embryos and regenerated plants. Consequently, we performed analysis of transcription profile of *tRNA*^*Glu*^ and *Sig2* genes whose expression changes are related to transition from NEP to PEP-dependent transcription. *Sig2*, located in nucleus, encodes a sigma factor necessary for transcription initiation of plastome genes by PEP. Among these genes is *tRNA*^*Glu*^*,* whose transcription product, after reaching a certain level, inhibits the activity of NEP in plastids [49]. Both genes: *Sig2* and *tRNA*^*Glu*^*,* showed a similar expression pattern during regeneration but different for both cultivars. In cv. ‘Jersey’ the expression levels of *Sig2* and *tRNA*^*Glu*^ genes were significantly higher than in cv. ‘Mercada’ at each time point of plant regeneration (Fig. 7). Already on 46dC the expression levels of *Sig2* and *tRNA*^*Glu*^ genes were 37 and 5 times higher than on 43dC in cv. ‘Jersey’, while they remained unchanged until 55dC in cv. ‘Mercada’. At this time point a remarkably high increase of *Sig2* and *tRNA*^*Glu*^ expression was observed in ‘Jersey’ (380 times and 20 times higher compared to 43dC, respectively). These results indicate that the transcription occurring in plastids of ‘Jersey’ embryos was dependent on PEP as early as embryo conversion stage, whereas at the same developmental stage in ‘Mercada’ embryos, the transcription was still predominantly carried by NEP.

### Chloroplast differentiation during regeneration of androgenic plants

The light-induced proplastid-to-chloroplast transition is a required step for further chloroplast differentiation that involves the efficient activation of expression of plastid genes, including *rRNA* genes, and induction of photomorphogenesis. Thus, during regeneration of androgenic plants we performed the analysis of expression of genes related to photomorphogenesis (genes encoding phytochromes and transcription factors that regulate photomorphogenesis), as well as genes involved in chloroplast differentiation, including regulation of chlorophyll synthesis, thylakoid synthesis and docking (Additional file 1: Table S1).

**Fig. 8:**
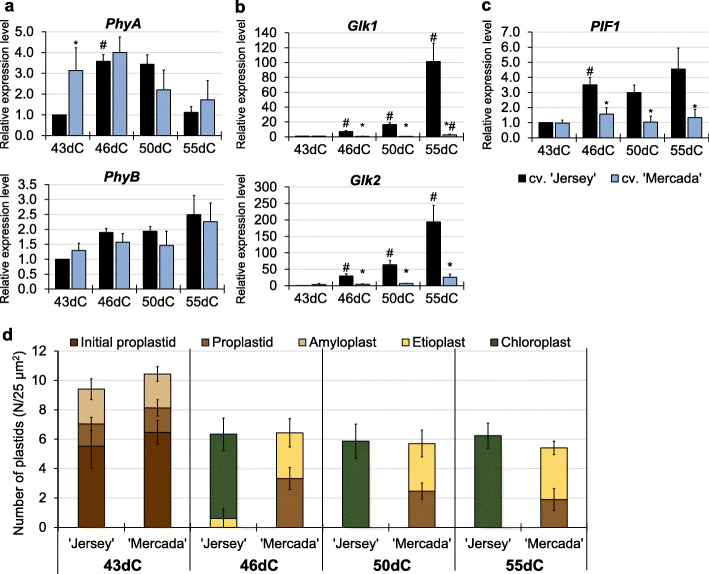
Chloroplast differentiation during regeneration of androgenic plants of ‘Jersey’ and ‘Mercada’ cultivars. **a**, **b**, **c** The relative expression level of nuclear-localised genes encoding: phytochromes A and B (**a**), transcription factors GLK1 and GLK2 (**b**) and PIF1 regulating the chlorophyll synthesis (**c**). **d** Types and density of plastids observed during regeneration. Graphs show mean values of *n ≥* 3 with SEM in **a-c** and SD in **d**. Relative expression level was normalised to 43dC of cv. ‘Jersey’. An asterisk presents a value significantly different between cultivars at a certain day of culture. A hash indicates a value significantly different from the preceding day of culture within cultivar (Tukey’s test, *P* < 0.05). dC – day of culture

No significant differences in expression profiles of *PhyA* and *PhyB* encoding phytochromes were observed between cvs. ‘Jersey’ and ‘Mercada’ during regeneration, except for androgenic embryos on 43dC (Fig. 8a). We observed, however, starting from 46dC, substantial differences between both cultivars in expression of *Glk1* and *Glk2* genes encoding transcription factors that are positive regulators of photosynthesis-associated nuclear genes. In cv. ‘Jersey’ there was a significant increase of both genes expression, reaching 100–200 times higher level on 55dC compared to 43dC (Fig. 8b). Contrary to ‘Jersey’, in cv. ‘Mercada’ expression of both *Glk* genes was constant during embryo conversion and plant regeneration. The high increase of *Glk* genes expression in cv. ‘Jersey’, observed already in converting embryos, indicates the activation of photomorphogenesis in ‘Jersey’ plastids in the GLK-dependent pathway. The lack of increased expression of *Glk* genes in cv. ‘Mercada’ suggests that chloroplast differentiation was detained during regeneration stage in this genotype.

A significant difference was observed in expression profile of *PIF1* regulating the chlorophyll synthesis which is also affected by light perception. In cv. ‘Jersey’ activation of *PIF1* expression was observed in converting embryos on 46dC, whereas in cv. ‘Mercada’ a twice lower expression compared to cv. ‘Jersey’ was observed, that was stable throughout the whole regeneration period (Fig. 8c). Such differences between cultivars were not observed in expression profiles of *Hy5* encoding another transcription factor involved in activation of nuclear genes controlling chloroplast development and *PGP1* and *RABA5e* genes involved in synthesis and docking of thylakoid membrane, respectively, which are not affected by light perception (Additional file 1: Fig. S4). PIF1 is a bHLH transcription factor, which is a critical modulator of cotyledon greening of dark-grown seedlings. PIF1 promotes seedling greening in two ways: first, it represses the accumulation of protochlorophyllide by regulating the expression of genes involved in the tetrapyrrole pathway; second, PIF1 directly binds to the promoter of PORC to activate its transcription, thus promoting the catalysis of protochlorophyllide into chlorophyll [68, 69].

Transmission electron microscopy analysis was performed to evaluate the number and describe types of plastids present in converting embryos and in leaves of regenerated plants on 43, 46, 50 and 55 day of culture of ‘Mercada’ and ‘Jersey’. No differences in plastid number per 25 μm^2^ of embryo cell cytoplasm between ‘Jersey’ and ‘Mercada’ were observed in the subsequent days of regeneration (Fig. 8d). Also, on 43dC, similar types of plastids (initial proplastids, proplastids and amyloplasts) were observed in similar proportion in both genotypes. However, since 46dC, when shoot apex become clearly visible in converting embryos, the significant differences were present in plastid types between analysed cultivars. In mesophyll cells of ‘Jersey’ regenerants, the majority of plastids were represented by chloroplasts, and only a small number of etioplasts was present on 46dC (Fig. 8d). Chloroplasts were characterised by well-developed grana, whereas etioplast contained only prolamellar body (Additional file 1: Fig. S5). Later on, on 50dC and 55dC, only chloroplasts were observed in leaves of ‘Jersey’ regenerants, 90% of which were green. On the other hand, in cv. ‘Mercada’ that regenerated mostly albino plants, on 46dC a similar fraction (50%) of proplastids and etioplast-like plastids was observed (Fig. 8d). Similar results were obtained for mesophyll cells of ‘Mercada’ regenerant leaves on 50dC and 55dC. The etioplast-like plastids in cv. ‘Mercada’ regenerants were more advanced in development and contained single perforated thylakoids and incipient grana without organized structure (Additional file 1: Fig. S5).

### Characterisation of albino regenerants of cvs. ‘Jersey’ and ‘Mercada’

Cv. ‘Jersey’, which in isolated microspore culture regenerated mostly green plants, produced also a small number (5–10%) of albino regenerants. To determine whether the processes that caused the albino formation in ‘Jersey’ were similar to those which occurred in ‘Mercada’, we compared expression profiles of genes involved in plastid biogenesis, chloroplast differentiation, photomorphogenesis and photosynthesis (Additional file 1: Table S1), as well as plastid ultrastructure and plastome replication in the albino and green regenerants of both genotypes.

**Fig. 9:**
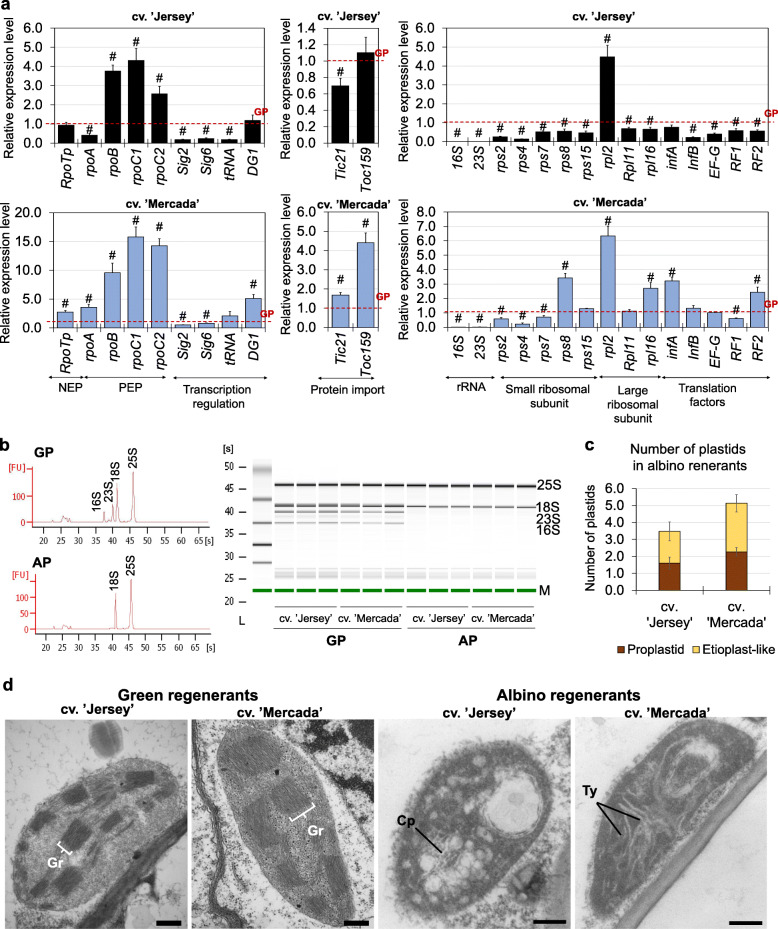
Comparison of albino and green regenerants of ‘Jersey’ and ‘Mercada’ cultivars. **a** The relative expression level of genes related to transcription, translation and protein import to plastids in albino regenerants of cvs. ‘Mercada’ and ‘Jersey’ compared to corresponding green regenerant. Graphs show mean values of *n ≥* 3 with SEM. Relative expression level was normalised to green regenerants of each cultivar. A hash indicates a value significantly different between green and albino plants within cultivar (t-Student test, *P* < 0.05). **b** Electropherograms and gel image of the RNAs isolated from green (GP) and albino (AP) regenerants showing nuclear and plastid rRNAs content using Agilent 2100 Bioanalyzer. **c** Types and number of plastids in single cross-section of mesophyll cell of albino regenerants of both cultivars. **d** Morphology of plastids observed in albino and green plants of cv. ‘Jersey’ and cv. ‘Mercada’. Graph shows mean values of *n* ≥ 100 with SD. Scale bars = 200 nm. Cp – prolamellar body, Gr – granum, Ty – thylakoid

The albino plants of both cultivars differed from the green regenerants of the corresponding cultivar in the expression profile of genes related to plastid transcription, translation and protein import to plastids (Fig. 9a). In albino regenerants of cv. ‘Jersey’ most of the tested genes engaged in these processes exhibited significantly lower expression compared to green regenerants. What is more, the relative expression levels of the genes that are associated with the transition from NEP to PEP transcription (*rpoA*, *Sig2*, *tRNA*^*Glu*^) were 2 to 5-fold lower, whereas the activity of genes encoding subunits of PEP (*rpoB*, *rpoC1* and *rpoC2*), that are predominantly transcribed by NEP, were significantly higher than in green regenerants. This data suggested that transcription occurring in plastids of ‘Jersey’ albino plants was not switched to the PEP-dependent process and the NEP was the leading plastid RNA polymerase, similarly to the plant regeneration in cv. ‘Mercada’ cultures.

Albino regenerants of cv. ‘Jersey’ and cv. ‘Mercada’ exhibited a very low level of the plastid rRNA transcripts which was visualised by quality control of RNA samples of green and albino regenerants using Agilent 2100 Bioanalyzer (Fig. 9b). However, contrary to cv. ‘Jersey’, the albino plants of cv. ‘Mercada’ showed a similar or even higher expression level of the majority of analysed genes (except for *tRNA*^*Glu*^, *Sig2*, *rRNAs*) than green regenerants of this cultivar (Fig. 9a). These results indicate that transcription of genes involved in plastid biogenesis was still ongoing in albino plants of cv. ‘Mercada’. Furthermore, when the albino regenerants of two cultivars were compared, the relative expression level of the majority of genes related to plastid biogenesis in cv. ‘Mercada’ was higher than in cv. ‘Jersey’, independently from the gene localisation in the plastid or nuclear genome (Additional file 1: Fig. S6). For example, RT-qPCR analysis revealed that the expression of *16S* and *23S rRNA* was two to four times lower in albino plants of cv. ‘Jersey’ than ‘Mercada’ (Additional file 1: Fig. S6).

**Fig. 10:**
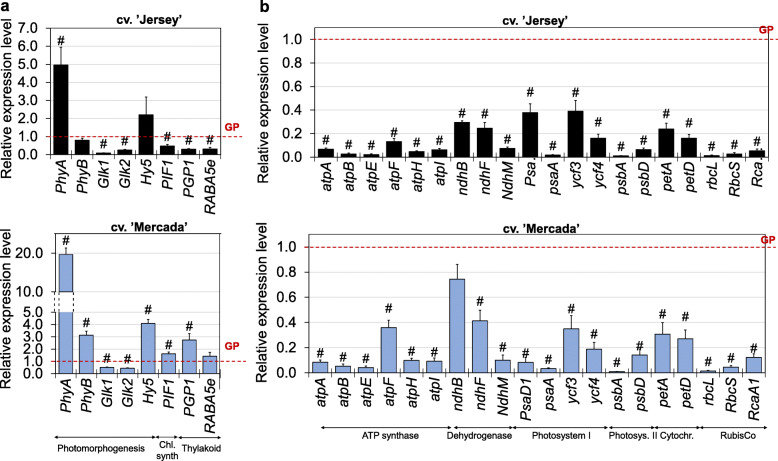
The relative expression of genes related to photomorphogenesis (**a**) and photosynthesis (**b**) in albino regenerants of 'Jersey' and 'Mercada' cvs. Graphs show mean values of *n ≥* 3 with SEM for relative expression level in albino plants normalised to green regenerants (GP) of corresponding cultivar. A hash indicates a value significantly different in albino plant from green regenerant (t-Student test, *P* < 0.05). *NdhM*, *PsaD1*, *RbcS* and *RcaA1* genes are encoded in the nuclear genome

Albino regenerants of both, ‘Jersey’ and ‘Mercada’ exhibited a significantly lower, than green plants, expression of genes encoding GLK transcription factors participating in regulation of photomorphogenesis. Also the transcription activity of other genes involved in chloroplast differentiation, such as *PIF1* (regulating the chlorophyll synthesis), *PGP1* (involved in synthesis of thylakoid membranes) and *RABA5e* (responsible for docking of thylakoid membranes) was reduced in albino regenerants of cv. ‘Jersey’ (Fig. 10). Interestingly, in albino regenerants of cv. Mercada’, the transcription level of all examined genes related to photomorphogenesis was similar or higher compared to the green plants. These results were in agreement with the observations of plastid ultrastructure in regenerants of both genotypes. TEM analysis of plastids present in leaf mesophyll cells of albino regenerants revealed differences between ‘Jersey’ and ‘Mercada’. In albino regenerants of both cultivars, we identified only differentiating proplastids and plastids similar to etioplasts, however at different stages of differentiation (Fig. 9c, d). That etioplast-like plastids in ‘Jersey’ contained only the prolamellar body, and no structures similar to prothylakoids were observed (Fig. 9d). Whereas in the case of etioplast-like plastids in ‘Mercada’, both the prolamellar body and non-organized prothylakoid/thylakoid structures were present (Fig. 9d). Since in both genotypes the conversion of observed plastids to chloroplast did not occurred, these plastids cannot be described as functional etioplasts. Contrary to albino plants, the structure of chloroplasts present in green regenerants did not differ between cultivars studied (Fig. 9d).

As a result of the failed plastid-to-chloroplast transition, albino regenerants of both, ‘Jersey’ and ‘Mercada’ exhibited significantly lower expression of plastid and nuclear genes that encode subunits of photosystems I and II, ATP synthase, cytochrome f, NADH-PQ oxidoreductase and RubisCo complex, compared to the green regenerants of a corresponding cultivar (Fig. 10).

**Fig. 11:**
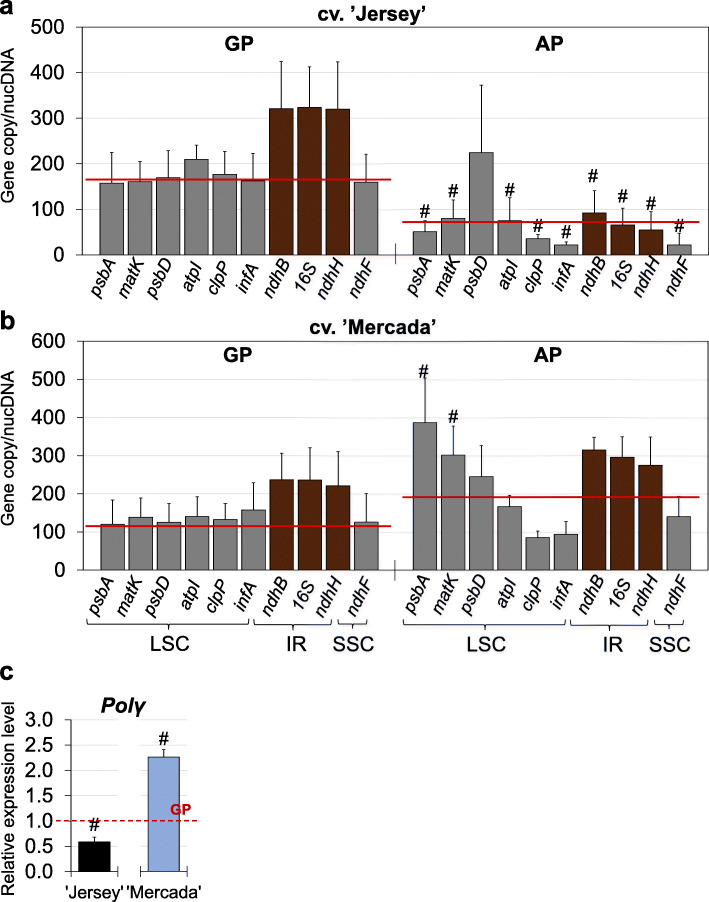
Plastid gene copy number in androgenic regenerants of ‘Jersey’ and ‘Mercada’ cultivars. **a**, **b** The individual copy number of genes localised in the plastid genome in green (GP) and albino (AP) regenerants of cv. ‘Jersey’ (**a**) and cv. ‘Mercada’ (**b**). **c** The relative expression profile of Polγ (Organellar DNA polymeraseI) gene in albino regenerants of both cultivars in comparison to green regenerants. Graphs show mean values of *n* ≥ 3 with SD in **a** and **b** and SEM in **c**. A hash indicates a value significantly different between green and albino plants within cultivar (t-Student test, *P* < 0.05). Red lines in **a** and **b** show average copy numbers calculated for copies of individual genes. LSC – long single copy, IR – inverted repeat, SSC – short single copy

In addition to gene expression and plastid ultrastructure, we assessed the copy number of plastom-localised genes in green and albino regenerants. In green plants of both cultivars, the copy number of individual genes was consistent with their localisation within the plastome. On the contrary, the albino plants of both cultivars contained various numbers of copies of individual genes, which were inconsistent with their predicted number based on gene location in the plastid genome (Fig. 11). In ‘Jersey’, the average plastome copy number was three times lower in albino plants, compared to green regenerants (Fig. 11a). Additionally, genes localised in inverted repeats of the plastid genome did not occur in the predicted double number of copies compared to genes located in single copy regions. This suggests that number of complete plastomes and thus templates were limited in albino plants of cv. ‘Jersey’, which in turn might result in the low expression level of plastidial *rRNA* genes, the arrest of chloroplast differentiation and albino phenotype of regenerants carrying these changes.

Contrary to ‘Jersey’, in albino regenerants of cv. ‘Mercada’ the average number of plastomes was two times higher than in green plants (Fig. 11b). As we observed already during plant regeneration (Additional file 1: Fig. S3b), the copy number of individual genes in ‘Mercada’ albino plants was not consistent with the value predicted on the basis of gene localisation. What was interesting, the genes *psbA* and *matK* localised proximal to the replication initiation site showed even three times higher number of copies in albino compared to green regenerants (Fig. 11b). The copy number of genes decreased along with the distance from the replication initiation site, which may indicate that replication has been often initiated, but not completed.

Additional support for this observation was provided by expression analysis of *Polγ* gene whose expression was two-fold higher in the albino than in green regenerants of cv. ‘Mercada’. The opposite expression pattern was observed for cv. ‘Jersey’, in which the expression of *Polγ* was two times higher in green than albino plants (Fig. 11c).

Comparison of cvs. ‘Jersey’ and ‘Mercada’ clearly showed that the cultivars presenting opposing ratio of green and albino regenerants showed the arrest of chloroplast differentiation at an early stage of biogenesis. This arrest was related to the lack of PEP-dependent transcription and inhibition of photomorphogenesis.

## Discussion

Vacuolated microspores treated with stress factors, e.g., starvation or cold, as applied in this study, can be induced in culture to change their developmental pathway and to induce microspore embryogenesis. It is well known that the reprogramming of microspore pathway from a gametophytic into a sporophytic one is possible only at a narrow window during microsporogenesis and in barley the efficient embryo induction is routinely initiated from ML (mid-to-late) uninucleate microspores [70, 71]. ML microspores of cvs. ‘Jersey’ and ‘Mercada’ used in our experiments went successfully through these reprogramming processes as they started mitotic divisions and formed embryos with a high frequency. Both cultivars exhibited a similar induction and regeneration potential of ca. 100 plants per 100,000 isolated microspores, however they differed significantly in their ability to produce green plants.

Regeneration of green plants depends on formation of chloroplasts which in androgenic plants originate from proplastids enclosed in microspores that initiate in vitro culture. Proplastids present in microspores are programmed to differentiate into amyloplasts, however as we have previously showed, the stage of microspore development at which activation and differentiation into amyloplast occurred, varied between barley cultivars. We demonstrated that the genotype ability to regenerate green plants in microspore culture depended on the molecular differentiation of microspore plastids prior to in vitro culture [18]. Cultivars producing mostly albino regenerants in androgenesis, such as ‘Mercada’, showed early activation of starch synthesis genes and early differentiation of proplastids into amyloplasts. At the ML uninucleate stage, routinely used for initiation of androgenic culture, amyloplasts filled with starch accounted for almost 50% of plastids present in the microspores of cv. ‘Mercada’, while in microspores of cv. ‘Jersey’ producing mostly green regenerants, only proplastids with no distinctive morphology were observed. Furthermore, in microspores of cv. ‘Mercada’ the activation of reserve starch synthesis genes occurred already at the early-mid (EM) stage of development, which precedes the stage used for culture initiation [18].

In the presented study we analysed plastid differentiation in microspores cultured in vitro. Using the same two barley cultivars ‘Jersey’ and ‘Mercada’ we examined the differentiation of plastids at the successive stages of in vitro culture, from the pre-treated microspores, through embryo induction and embryo formation phase to the plant regeneration. As mentioned above, in the ML microspores of cv. ‘Mercada’ almost a half of proplastids have already been differentiated into amyloplast, which were not present in ML microspores of ‘Jersey’. Since the fourth day of culture (4dC) we identified the appearance of starch-accumulating plastids also in dividing microspores and microspore-derived structures of cv. ‘Jersey’. However, the analysis of gene expression revealed a different function of these amyloplasts compared to ‘Mercada’. The expression of genes encoding enzymes of reserve starch synthesis was markedly reduced, while the genes responsible for assimilatory starch synthesis were activated in ‘Jersey’ at the beginning of in vitro culture. The physiological function of reserve and assimilatory starch differs: while the reserve starch is stored for the long period and used as a source of energy in the next generation, the assimilatory starch is temporarily accumulated during the day and is rapidly consumed to support biological activities [72]. In both cultivars, the number of amyloplasts and expression of assimilatory starch synthesis genes reached the highest values on 21dC, when pro-embryo have been formed.

It should be noted that the increased number of amyloplasts in cv. ‘Mercada’ was observed before activation of genes related to the assimilatory starch synthesis. Therefore, we assumed that the majority of amyloplasts present in ML microspores of ‘Mercada’ were not degraded after pre-treatment and culture initiation, and could divide as it was described in starch accumulating tissues, for example in *Oryza sativa* and *Colocasia esculenta* [73, 74]. Thus, the applied pre-treatment did not reverse the process of plastid differentiation in ML microspores of cv. ‘Mercada’, including starch synthesis and accumulation. However, the pre-treatment resulted in a decrease or even disappearance of differences between both cultivars in expression profile of most genes related to plastid transcription and translation. It also hindered the process of plastome degradation that occurs during pollen grain formation in vivo*.* The degradation of plastomes observed in ‘Mercada’ microspores was correlated in this study with the activation of expression of *DPD1* exonuclease responsible for degradation of plastid DNA during pollen development [66]. In ‘Mercada’ *DPD1* was expressed throughout microsporogenesis, since the early stage of microspore development, which was not observed in cv. ‘Jersey’. During successive stages of in vitro culture both cultivars exhibited a low expression of *DPD1* and a changing number of plastomes.

The number of plastid copies increased during embryo induction and formation phase in both genotypes and reached the highest value on 21dC, in accordance with the highest number of plastids observed at this time point.

Early phase of chloroplast differentiation is regulated by checkpoints at proplastid development, including the stability of plastomes and effective transcription and translation processes occurring in proplastids [54, 75]. Decrease of the number of plastid genomes is frequently observed during chloroplast differentiation associated with plant development [76]. High demand for plastidial mRNAs and rRNAs within the cell is covered by the great number of plastids and many copies of plastomes within plastid [77, 78]. We observed a significant decline of the plastome copy number in both cultivars between 21dC and 35dC, i.e. during embryo differentiation phase. In cv. ‘Jersey’, in contrast to cv. ‘Mercada’, the plastome copy number increased during the next phase of embryo development, i.e. embryo body axis formation. Additionally, in cv. ‘Mercada’ since 35dC the copy numbers of individual genes differed from the values expected on the basis of gene localisation within plastome. During conversion of cv. ‘Mercada’ embryos and further development of regenerants, the average copy number of plastomes increased, however the number of copies of individual genes were even more divergent. This indicates that the copies of replicating plastome were incomplete compared to the full copies. The high number of proper plastid genomes is considered as a checkpoint for chloroplast differentiation [79]. It was shown in Arabidopsis that the instability of plastid genomes blocked differentiation of proplastid into chloroplast [80, 81]. Plastid genome of cv. ‘Mercada’ during androgenic culture showed high instability. Thus, we assume that that the low number of correct plastome copies could influence the differentiation of proplastids into chloroplasts in cv. ‘Mercada’.

The transition from NEP- to PEP-dependent transcription in plastids is a crucial factor in chloroplast differentiation, as only plastidial transcription system is capable of providing a high level of transcripts of plastid-encoded genes including rRNAs, tRNAs, genes for some ribosomal proteins involved in plastid translation, as well as genes encoding subunits of photosystems [42, 82]. Mutation in each of PEP subunit genes caused an albino phenotype and a lack of photosynthesis in tobacco (*Nicotiana tabacum* L.) [83, 84], while a knock-out of *RpoTp* (encoding NEP) in Arabidopsis resulted in delayed chloroplast development only [85]. The progress of chloroplast differentiation, which involves light, requires SIG2-dependent transcription of plastid genes carried out by PEP. Among these genes is the *tRNA*^*Glu*^ gene whose transcription product, after reaching a certain level, inhibits the activity of NEP and thus serves as a checkpoint for induction of chloroplast differentiation [47, 49, 50, 86]. In addition, the charged glutamyl-tRNA is the precursor for 5-aminolevulinic acid whose level correlates with control of nuclear genes mediated by retrograde signalling [47, 48].

Expression profiling of *Sig2* and *tRNA*^*Glu*^ revealed significant differences between ‘Jersey’ and ‘Mercada’ cultivars, indicating different transcription activity of polymerases NEP and PEP. In cv. ‘Jersey’ the activation of PEP-depended transcription was observed already in converting embryos on 46dC. At this time point, the relative expression levels of *Sig2* and *tRNA*^*Glu*^ genes in differentiating ‘Jersey’ embryos increased 37-fold and 5-fold, respectively, compared to their levels on 43dC. Contrary to ‘Jersey’, the PEP-dependent transcription during early stages of plant regeneration in cv. ‘Mercada’ was not observed. The relatively low expression level of *tRNA*^*Glu*^ throughout plant regeneration indicates that NEP was still the dominant RNA polymerase in ‘Mercada’ plastids. The additional support for ongoing activity of NEP in regenerating plants of cv. ‘Mercada’ was given by a high expression of *rpoB* gene that is dominantly transcribed by NEP. Furthermore, expression profile of *Sig2* normalised to the reference genes *ARF1* (*ADP-ribosylation factor 1-like protein*) and *EF1* (*Translation elongation factor 1-a*), clearly showed that this nuclear gene was active only since 46dC and only in ‘Jersey’ embryos (Additional file 1: Fig. S7). This raises the question about the factor (factors) that triggered the *Sig2* activation in ‘Jersey’ embryos but did not act in ‘Mercada’. We speculate that the mechanism leading to the lack of *Sig2* expression in ‘Mercada’ may be related to the plastid genome instability observed during embryo formation in this cultivar. As described above, since 35dC, the plastids of cv. ‘Mercada’ contained incorrect copies of plastid genomes and on 43dC they had the lowest number of genome copies during the whole in vitro culture. The integrity of plastid genome is considered as a checkpoint during early proplastid-to-chloroplast differentiation [87, 88]. Differentiation of chloroplasts depends on the effective nucleus-to-plastid (anterograde) and plastid-to-nucleus (retrograde) signaling and the lack of signal from plastids might result in the absence or deficiency of transcription activation in the nucleus. Nevertheless, the molecular mechanisms underlying the retrograde signal that activates *Sig2* gene in the nucleus remain to be uncovered.

The role of RNA polymerases seems to be crucial in regenerating green plants in microspore embryogenesis as the albinism is a phenomenon occurring solely in cereals that harbour only one nuclear-encoded polymerase (*RpoTp*) [51]. Dicots, that do not regenerate albino plants in androgenic culture, contain two nuclear-encoded polymerases: *RpoTp* and *RpoTmp*. The latter is active mostly in mitochondria [51] but it also participates in transcription of *16S rRNA* occurring in plastids [40, 89]. The main consequence of the failed NEP-to-PEP transition in ‘Mercada’ plastids was the lack of activation of efficient rRNAs transcription by PEP during embryo conversion and plant regeneration stages. In ‘Jersey’ embryos, where this transition took place, the relative expression of *16S* and *23S* genes encoding plastid rRNAs increased 20 to 30-fold between 43dC and 46dC and reached 300–500 times higher level in the regenerated plantlets on 55dC. When expression profiles of plastid *rRNA* genes normalised to the reference genes are observed throughout the whole androgenesis process, it is clearly seen that the increase in rRNA levels took place only in cv. ‘Jersey’ and was initiated on 43dC (Additional File 1: Fig. S7). At this time point, when body axis in embryos became visible, embryo had been cultured for 8 days on regeneration medium, first at darkness and for 3 days in light. In contrast to ‘Jersey’, during regeneration of cv. ‘Mercada’ plants we did not observe any significant increase in the level of plastid-encoded rRNA transcripts between 43dC and 55dC. The 16S and 23S rRNAs are required for ribosome assembling and their lack results in ribosome depletion [53], while the proper translation occurring in plastids is necessary for induction of proplastid-to-chloroplast differentiation [90].

As a consequence of incorrect plastid biogenesis, genes encoding transcriptional factors GLKs, that are positive regulators of photosynthesis-associated nuclear genes, as well as genes related to photosynthesis were not activated in regenerating ‘Mercada’ embryos and albino regenerants of both cultivars. Similarly, barley *albostrians* mutant with depletion in plastidial ribosomes showed the reduced content of mRNAs for photosynthesis-related proteins [91].

Continuous activity of NEP in albino regenerants of cv. ‘Mercada’ resulted in accumulation of transcripts of plastid genes whose transcription is NEP-dependent. Also other genes involved in plastid biogenesis, such as import (*Tic21*, *Toc159*) and plastome replication stayed active. The increased expression of these genes indicate the response at the transcription level in order to regain plastid biogenesis and maintain chloroplast differentiation. Studies in Arabidopsis showed that the low activity of PEP in plastids, caused by depletion of PEP subunits or sigma factors, resulted in the increased transcription of genes dependent on NEP [47, 84, 92]. The increased level of PEP subunit transcripts encoded by *rpoA*, *rpoB*, *rpoC1*, *rpoC2* genes was also revealed in barley *albostrians* and maize *iojap* mutants that lack plastid 70S ribosomes, as well as in most other albino mutants [42, 93, 94].

Interestingly, plastids in mesophyll cells of albino plants of cv. ‘Mercada’ were more advanced in differentiation than in albino plants of cv. ‘Jersey’ that occasionally appeared among regenerants. TEM observations showed the presence of both, the prolamellar body and non-organized prothylakoid/thylakoid structures in the etioplast-like plastids of ‘Mercada’, while in ‘Jerey’ etioplasts only prolamellar bodies were present. Albino plants of cv. ‘Jersey’ exhibited a 2-fold lower average number of plastome copies in comparison to green regenerants and a significant deviation between numbers of individual gene copies. The low number of plastidial gene copies, including rRNA-encoding genes, provided a limited number of templates for transcription occurring in plastids, which in turn impeded the light-dependent chloroplast biogenesis and resulted in the arrest of plastids at early stage of differentiation. The various number of copies of specific genes observed in albino plants of cv. ‘Jersey’ might result from incomplete plastome replications and/or structural changes in plastid genome, as described in albino regenerants of many cereals [19–22].

Based on the comparison of green and albino regenerants of cvs. ‘Jersey’ and ‘Mercada’ it is worth noting that the time of activation rather than the level of expression of specific genes alone is crucial in regeneration of green plants. Expression of many of analysed genes including *tRNA*^*Glu*^, *Sig2*, *Glk1*, *Glk2* increased in cv. ‘Mercada’ on 55th day of culture when the plants were already regenerated, yet the plastids observed in mesophyll cells of albino regenerants were already arrested at the early stage of development. The lack of the proper plastid biogenesis during embryo differentiation stage, discussed earlier, resulted in the lack of proplastid-to-chloroplast transition and regeneration of albino plants.

It should be underlined that the presented here mechanism leading to formation of albino plants in androgenic culture is a consequence of plastid differentiation during pollen development in vivo. The proplastids, which initiated the programme of proplastid-to-amyloplast differentiation, cannot be reversed by in vitro conditions. Therefore, microspores that contain such plastids at the stage of culture initiation will produce mostly albino regenerants in androgenesis. Additional support for this notion was provided by the induction of isolated microspore culture from the earlier stage of microspore development than routinely utilised. When microspores harbour only proplastids, it is possible to significantly increase the frequency of green regenerants in barley androgenesis [18].

## Conclusions

The study provides insights into molecular processes that lead to the formation of albino regenerants during cereal androgenesis. We showed that the failed transition from NEP-dependent to PEP-dependent transcription in proplastids present in microspore-derived embryos is associated with the impaired chloroplast differentiation during regeneration of androgenic plants, the inhibition of photomorphogenesis and the genotype inability to regenerate green plants. In the genotype that regenerated mostly albino plants, the very low level of 16S and 23S rRNA transcripts and the lack of *Sig2* expression in regenerating embryos indicated the failed activation of PEP RNA polymerase. The lack of the PEP activity in transcription of plastid-encoded genes, associated with the lack of *Sig2* gene activation in nucleus, indicates that proplastids did not pass the early checkpoint of their development. We suggest that the insufficient number of complete plastome copies, observed as early as differentiating embryos, might be a retrograde signal that prevented the correct plastid biogenesis and chloroplast differentiation in the albino-producing genotype.

## Methods

### Plant material and growth conditions

Two spring barley cultivars ‘Jersey’ and ‘Mercada’ were utilised to perform the experiments. ‘Jersey’ is used as a malting, whereas ‘Mercada’ is a fodder cultivar. Both cultivars are two-row. The seeds of both barley cultivars were provided by DANKO Plant Breeding Ltd., Poland.

The donor plants for isolated microspore in vitro cultures were sown and grown at 18/16°C, illumination 200 μM s^− 1^·m^− 2^ photon flux for 3 weeks in a growth room, followed by transfer to a growth chamber under controlled conditions at 17/14°C (day/night) temperature, illumination 480–500 μM s^− 1^·m^− 2^ photon flux and 16/8 h photoperiod.

### Isolated microspore in vitro culture and sampling

Spikes containing microspores at the mid-late to late (ML) developmental stage were collected to initiate the in vitro cultures and surface sterilised with 70% ethanol. The microspores were freshly isolated according to Coronado et al. [95]. The procedure was previously described in Gajecka et al. [18]. Briefly, ten spikes were homogenised twice for 20 s using a Waring Variable-Speed Laboratory Blender (Waring Laboratory Science) in 20 ml 0.4 M mannitol. The obtained homogenate was filtered through 100 μm nylon mesh. The microspores were collected by centrifugation (110×g; 10 min; 4°C) and suspended in 5 ml 0.55 M maltose overlaid with 2 ml 0.4 M mannitol and centrifuged (110×g; 10 min; 4°C). The viable microspores present in the interphase were collected and pre-treated in SMB1 medium [95] at 25°C for 48 h, in a density of 100,000 microspores per 1 ml medium in Petri dish. The medium was then exchanged for KBP induction medium [96] (Additional file 1: Table S2). The cultures were incubated at 25°C in the dark for 7 days. Next, one ml of fresh KBP medium was added and the culture incubation was continued under the same conditions on a rotary shaker at 65 rpm for another 14 days. The developed multicellular structures on 21st day were transferred onto KBPD differentiation medium [95] (Additional file 1: Table S2) and cultured at 25°C in the dark. After 2 weeks, the microspore-derived embryos on 35th day of culture were placed on K4NB regeneration medium [96] (Additional file 1: Table S2) and kept at 25°C in the dark for 5 days, followed by exposition to 100 μM s^− 1^·m^− 2^ of light with a 16/8 h photoperiod.

Separate microspore isolations in three independent biological replications were used to collect samples on the day of culture initiation (microspores at ML stage), SMB1 pre-treated microspores (PM), and successive days of culture induction phase including 2nd, 4th, 7th,21st day of in vitro culture, along with the end of embryos differentiation phase on 35th day and developed androgenic embryos on 43rd day. After embryo formation, during regeneration of androgenic plants the samples were collected at the time of embryos conversion on 46th day, and developing plants on 50th and 55th day of in vitro culture. To compare regenerants of both cultivars, the green and albino plants of cvs. ‘Mercada’ and ‘Jersey’ were sampled on 65th day of culture.

### Gene expression profiling using RT-qPCR

The total RNA was extracted in three independent biological repetitions using a ‘mirVana™ miRNA Isolation Kit’ (Thermo Fisher Scientific) according to the manufacturer’s instructions preceded by grinding samples in a frozen mortar. Isolated samples were evaluated using an ND-1000 spectrophotometer (Thermo Fisher Scientific) and Agilent RNA 6000 Nano Kit (Agilent Technologies) together with Bioanalyzer 2100 (Agilent Technologies). The RT-qPCR analysis was performed as was previously described by Gajecka et al. [18]. Briefly, one μg of total RNA per sample was treated with RQ1 RNase-Free DNase (Promega) and reverse transcribed according to the manufacturer’s instructions in a 20 μl reaction volume using a RevertAid First Strand cDNA Synthesis Kit (Thermo Fisher Scientific) with random primers. The cDNA was diluted five-fold with water and used at a volume of 2.5 μl in RT-qPCR. The reaction was carried out in a 10 μl volume using a LightCycler® 480 SYBR Green I Master (Roche) in two technical repeats. The primers used in the analysis (Additional file 1: Table S1) were designed with Primer3 [97]. Analysis was performed using a LightCycler 480 (Roche) under the following reaction conditions: initial denaturation 5 min at 95°C, followed by 10 s at 95°C, 20 s at a temperature specific for the primers, 10 s at 72°C, repeated in 40 cycles. Denaturation for the melt curve analysis was conducted for 5 s at 95°C, followed by 1 min at 65°C and heating up to 98°C (0.1°C/s for the fluorescence measurement). The Ct values and the value of the qPCR efficiency were obtained from LinRegPCR [98] and used for calculations. The relative expression level was calculated using the ΔΔCt method [99] and calibrated to the ML microspores, embryos on 43rd day of culture or regenerants of cv. ‘Jersey’ as stated in figures descriptions. As an internal control, two genes, *ARF1* and *EF1* were used preceded by the evaluation of the stability of expression using NormFinder [100] and BestKeeper [101]. To estimate the significant differences (at *P* < 0.05) between the compared samples, the One Way Analysis of Variance followed by Tukey’s HSD test was applied unless otherwise noted.

### Estimation of plastid gene copy number

In order to extract DNA, collected tissues were homogenised in liquid nitrogen with glass beads (Sigma Aldrich) using a FastPrep Instrument (MP Biochemicals) followed the C-TAB method [102]. The isolated DNA was treated with 10 μg of RNase at 37°C for 45 min. The concentration and purity of the isolated samples were evaluated using an ND-1000 spectrophotometer (Thermo Fisher Scientific). 50 ng of DNA was used as the template to quantify the gene copy number in a 10 μl volume using a LightCycler® 480 SYBR Green I Master (Roche) in two technical repeats in LightCycler 480 (Roche) under conditions described above. The primers used in the analysis (Additional file 1: Table S2) were designed with Primer3 [97]. The Ct values and the value of the qPCR efficiency were obtained from LinRegPCR [98] and used for calculations. The plastid genes were quantified as previously described [18] in relation to two single copy nuclear genes: *ARF1* and *EF1* (Additional file 1: Table S2). The quantified genes were localised within a plastid genome as followed: *psbA*, *matK*, *psbD*, *atpI*, *clpP*, *infA* in the long single copy region (LSC); *ndhB*, *16S*, *ndhH* in the inverted repeat region (IR) and *ndhF* in the short single copy region (SSC, Additional file 1: Table S2). Plastome genes located in the IRs are present in two copies. The values obtained for each of plastid gene were used to calculate the average plastome copy number. To estimate the significant differences (at *P* < 0.05) between the compared samples, the One Way Analysis of Variance followed by Tukey’s HSD test or t-Student test were applied as mentioned in the figure description.

### Transmission electron microscope (TEM) analysis

An electron microscopic analysis was performed using a Tecnai Sphera G2 (FEI Company), as previously described [18]. Briefly, the plant material was fixed by immersion in a 50 mM cacodylate buffer (pH 7.2) for 6 h at RT, then it was washed in a cacodylate buffer and twice in distilled water. The cacodylate buffer contained 0.5% (v/v) glutaraldehyde and 2.0% (v/v) formaldehyde. Next, the samples were fixed in 1.0% (v/v) osmium tetroxide for 1 h at RT, washed twice in distilled water, dehydrated by passage through an acetone series (20–100%) and infiltrated with Spurr resin (Sigma Aldrich) initially 33%, then 66% and finally 100%.

The analysis was performed in three independent biological repetitions with at least 100 cells per repetition. The types of plastids were recognised according to the common description as initial undifferentiated proplastids, differentiating proplastids with few internal membranes and dense matrix, amyloplasts, etioplasts and chloroplasts.

## Supplementary Information

**Additional file 1: Fig. S1.** Expression profiles of genes related to translation occurring in plastid during isolated microspore culture of cvs. ‘Jersey’ and ‘Mercada’. **Fig. S2.** The relative expression profile of *DPD1* gene encoding Mg^2 + ^- dependent organelle exonuclease during microspore development of cvs. ‘Jersey’ and ‘Mercada’. **Fig. S3.** Plastid DNA content during regeneration of androgenic plants of cvs. ‘Jersey’ and ‘Mercada’. **Fig. S4.** The expression profiles of genes related to chloroplast differentiation during regeneration of plants of cvs. ‘Jersey’ and ‘Mercada’. **Fig. S5.** The plastids observed in converting embryos on 46dC of cvs. ‘Jersey’ and ‘Mercada’. **Fig. S6.** The relative expression level of genes related to plastid biogenesis, chloroplast differentiation and photosynthesis in albino regenerants of cv. ‘Mercada’ compared to albino regenerants of cv. ‘Jersey’. **Fig. S7.** The normalized expression level of genes important for plastid biogenesis during embryo formation and regeneration of androgenic plants. **Table S1.** List of genes and primers used to perform RT-qPCR analysis. **Table S2.** Composition of media used in isolated microspore culture. **Table S3.** List of genes, genome localisation and primers used to evaluate plastid DNA copy number using qPCR.

## Data Availability

All data generated or analysed during this study are included in this published article and its supplementary information files. The raw datasets generated during the current study are available from the corresponding author on reasonable request.

## Abbreviations

dC: Day of culture
ML: Mid-to-late microspore
NEP: Nuclear-encoded polymerase
PEP: Plastid-encoded polymerase
PM: Pre-treated microspores

## References

[1] Ferrie AMR, Caswell KL (2011). Isolated microspore culture techniques and recent progress for haploid and doubled haploid plant production. Plant Cell Tissue Organ Cult.

[2] Dwivedi SL, Britt AB, Tripathi L, Sharma S, Upadhyaya HD, Ortiz R (2015). Haploids: constraints and opportunities in plant breeding. Biotechnol Adv.

[3] Germanà MA (2011). Gametic embryogenesis and haploid technology as valuable support to plant breeding. Plant Cell Rep.

[4] Honys D, Reňák D, Twell D (2006). Male gametophyte development and function. Plant Biotechnol.

[5] Carrizo García C, Nepi M, Pacini E (2017). It is a matter of timing: asynchrony during pollen development and its consequences on pollen performance in angiosperms—a review. Protoplasma..

[6] Silva TD. Microspore Embryogenesis. In: Sato K-I, editor. Embryogenesis. InTech; 2012. p. 573–596.

[7] Islam SMS, Tuteja N (2012). Enhancement of androgenesis by abiotic stress and other pretreatments in major crop species. Plant Sci.

[8] Shariatpanahi ME, Bal U, Heberle-Bors E, Touraev A (2006). Stresses applied for the re-programming of plant microspores towards in vitro embryogenesis. Physiol Plant.

[9] Holme IB, Olesen A, Hansen NJP, Andersen SB (1999). Anther and isolated microspore culture response of wheat lines from northwestern and eastern Europe. Plant Breed.

[10] Lantos C, Páricsi S, Zofajova A, Weyen J, Pauk J (2006). Isolated microspore culture of wheat (*Triticum aestivum* L.) with Hungarian cultivars. Acta Biol Szeged.

[11] He T, Yang Y, Tu SB, Yu MQ, Li XF (2006). Selection of interspecific hybrids for anther culture of indica rice. Plant Cell Tissue Organ Cult.

[12] Marchand S, Fonquerne G, Clermont I, Laroche L, Huynh TT, Belzile FJ (2008). Androgenic response of barley accessions and F1s with *Fusarium* head blight resistance. Plant Cell Rep.

[13] Makowska K, Oleszczuk S, Zimny A, Czaplicki A, Zimny J (2015). Androgenic capability among genotypes of winter and spring barley. Plant Breed.

[14] Lantos C, Bóna L, Boda K, Pauk J (2014). Comparative analysis of *in vitro* anther- and isolated microspore culture in hexaploid Triticale (X *Triticosecale* Wittmack) for androgenic parameters. Euphytica..

[15] Clement C, Pacini E (2001). Anther plastids in angiosperms. Bot Rev.

[16] Caredda S, Devaux P, Sangwan RS, Clement C (1999). Differential development of plastids during microspore embryogenesis in barley. Protoplasma..

[17] Makowska K, Oleszczuk S (2014). Albinism in barley androgenesis. Plant Cell Rep.

[18] Gajecka M, Marzec M, Chmielewska B, Jelonek J, Zbieszczyk J, Szarejko I (2020). Plastid differentiation during microgametogenesis determines green plant regeneration in barley microspore culture. Plant Sci.

[19] Day A, Ellis THN (1985). Deleted forms of plastid DNA in albino plants from cereal anther culture. Curr Genet.

[20] Dunford R, Walden RM (1991). Plastid genome structure and plastid-related transcript levels in albino barley plants derived from anther culture. Curr Genet.

[21] Mouritzen P, Holm PB (1994). Chloroplast genome breakdown in microspore cultures of barley (*Hordeum vulgare* L.) occurs primarily during regeneration. J Plant Physiol.

[22] Mozgova GV, Zaitseva OI, Lemesh VA (2012). Structural changes in chloroplast genome accompanying albinism in anther culture of wheat and triticale. Cereal Res Commun.

[23] Day A, Ellis THN (1984). Chloroplast DNA deletions associated with wheat plants regenerated from pollen: possible basis for maternal inheritance of chloroplasts. Cell..

[24] Harada T, Sato T, Asaka D, Matsukawa I (1991). Large-scale deletions of rice plastid DNA in anther culture. Theor Appl Genet.

[25] Yamagishi M (2002). Heterogeneous plastid genomes in anther culture-derived albino rice plants. Euphytica..

[26] Ankele E, Heberle-Bors E, Pfosser MF, Hofinger BJ (2005). Searching for mechanisms leading to albino plant formation in cereals. Acta Physiol Plant.

[27] Chen XW, Cistué L, Muñoz-Amatriaín M, Sanz M, Romagosa I, Castillo AM (2007). Genetic markers for doubled haploid response in barley. Euphytica..

[28] Muñoz-Amatriaín M, Castillo AM, Chen XW, Cistué L, Vallés MP (2008). Identification and validation of QTLs for green plant percentage in barley (*Hordeum vulgare* L.) anther culture. Mol Breed.

[29] Krzewska M, Czyczyło-Mysza I, Dubas E, Gołębiowska-Pikania G, Żur I (2015). Identification of QTLs associated with albino plant formation and some new facts concerning green versus albino ratio determinants in triticale (×*Triticosecale* Wittm.) anther culture. Euphytica.

[30] Sakamoto W, Miyagishima S, Jarvis P (2008). Chloroplast biogenesis: control of plastid development, protein import, Division and Inheritance. Arab B.

[31] Pogson BJ, Ganguly D, Albrecht-Borth V (1847). Insights into chloroplast biogenesis and development. Biochim Biophys Acta Bioenerg.

[32] Bock R, Bock R (2007). Structure, function, and inheritance of plastid genomes. Cell and molecular biology of plastids.

[33] Nakai M. The TIC complex uncovered: The alternative view on the molecular mechanism of protein translocation across the inner envelope membrane of chloroplasts. Biochim Biophys Acta Bioenerg 2015;1847:957–967. 10.1016/j.bbabio.2015.02.011 25689609

[34] Belcher S, Williams-Carrier R, Stiffler N, Barkan A (2015). Large-scale genetic analysis of chloroplast biogenesis in maize. Biochim Biophys Acta Bioenerg.

[35] Barajas-López JDD, Blanco NE, Strand Å (2013). Plastid-to-nucleus communication, signals controlling the running of the plant cell. Biochim Biophys Acta.

[36] Inaba T (2010). Bilateral communication between plastid and the nucleus: plastid protein import and plastid-to-nucleus retrograde signaling. Biosci Biotechnol Biochem.

[37] Jarvis P (2008). Targeting of nucleus-encoded proteins to chloroplasts in plants. New Phytol.

[38] Flores-Pérez Ú, Jarvis P (2012). Molecular chaperone involvement in chloroplast protein import. Biochim Biophys Acta.

[39] Liebers M, Grübler B, Chevalier F, Lerbs-Mache S, Merendino L, Blanvillain R (2017). Regulatory shifts in plastid transcription play a key role in morphological conversions of plastids during plant development. Front Plant Sci.

[40] Yagi Y, Shiina T (2014). Recent advances in the study of chloroplast gene expression and its evolution. Front Plant Sci.

[41] Zhelyazkova P, Hammani K, Rojas M, Voelker R, Vargas-Suárez M, Börner T (2012). Protein-mediated protection as the predominant mechanism for defining processed mRNA termini in land plant chloroplasts. Nucleic Acids Res.

[42] Börner T, Aleynikova AY, Zubo YO, Kusnetsov VV (1847). Chloroplast RNA polymerases: role in chloroplast biogenesis. Biochim Biophys Acta Bioenerg.

[43] Pfannschmidt T, Blanvillain R, Merendino L, Courtois F, Chevalier F, Liebers M (2015). Plastid RNA polymerases: orchestration of enzymes with different evolutionary origins controls chloroplast biogenesis during the plant life cycle. J Exp Bot.

[44] Chi W, He B, Mao J, Jiang J, Zhang L (2015). Plastid sigma factors: their individual functions and regulation in transcription. Biochim Biophys Acta Bioenerg.

[45] Oh S, Montgomery BL (2013). Phytochrome-induced SIG2 expression contributes to photoregulation of phytochrome signalling and photomorphogenesis in Arabidopsis thaliana. J Exp Bot.

[46] Schweer J, Türkeri H, Kolpack A, Link G (2010). Role and regulation of plastid sigma factors and their functional interactors during chloroplast transcription - recent lessons from Arabidopsis thaliana. Eur J Cell Biol.

[47] Woodson JD, Perez-Ruiz JM, Schmitz RJ, Ecker JR, Chory J (2013). Sigma factor-mediated plastid retrograde signals control nuclear gene expression. Plant J.

[48] Czarnecki O, Gläßer C, Chen JG, KFX M, Grimm B (2012). Evidence for a contribution of ALA synthesis to plastid-to-nucleus signaling. Front Plant Sci.

[49] Hanaoka M, Kanamaru K, Fujiwara M, Takahashi H, Tanaka K (2005). Glutamyl-tRNA mediates a switch in RNA polymerase use during chloroplast biogenesis. EMBO Rep.

[50] Kanamaru K, Nagashima A, Fujiwara M, Shimada H, Shirano Y, Nakabayashi K (2001). An Arabidopsis sigma factor (SIG2)-dependent expression of plastid-encoded tRNAs in chloroplasts. Plant Cell Physiol.

[51] Liere K, Weihe A, Börner T (2011). The transcription machineries of plant mitochondria and chloroplasts: composition, function, and regulation. J Plant Physiol.

[52] Tiller N, Weingartner M, Thiele W, Maximova E, Schöttler MA, Bock R (2012). The plastid-specific ribosomal proteins of Arabidopsis thaliana can be divided into non-essential proteins and genuine ribosomal proteins. Plant J.

[53] Tiller N, Bock R (2014). The translational apparatus of plastids and its role in plant development. Mol Plant.

[54] Pfannschmidt T (2010). Plastidial retrograde signalling - a true “plastid factor” or just metabolite signatures?. Trends Plant Sci.

[55] Kobayashi Y, Kanesaki Y, Tanaka A, Kuroiwa H, Kuroiwa T, Tanaka K (2009). Tetrapyrrole signal as a cell-cycle coordinator from organelle to nuclear DNA replication in plant cells. Proc Natl Acad Sci.

[56] Jarvis P, López-Juez E (2013). Biogenesis and homeostasis of chloroplasts and other plastids. Nat Rev Mol Cell Biol.

[57] Fitter DW, Martin DJ, Copley MJ, Scotland RW, Langdale JA (2002). *GLK* gene pairs regulate chloroplast development in diverse plant species. Plant J.

[58] Hernández-Verdeja T, Strand Å (2018). Retrograde signals navigate the path to chloroplast development. Plant Physiol.

[59] Larkin R (2014). Influence of plastids on light signalling and development. Philos Trans R Soc B.

[60] Kobayashi K, Sasaki D, Noguchi K, Fujinuma D, Komatsu H, Kobayashi M (2013). Photosynthesis of root chloroplasts developed in Arabidopsis lines overexpressing *GOLDEN2*-*LIKE* transcription factors. Plant Cell Physiol.

[61] Nakamura H, Muramatsu M, Hakata M, Ueno O, Nagamura Y, Hirochika H (2009). Ectopic overexpression of the transcription factor *OsGLK1* induces chloroplast development in non-green rice cells. Plant Cell Physiol.

[62] Solymosi K, Schoefs B (2010). Etioplast and etio-chloroplast formation under natural conditions: the dark side of chlorophyll biosynthesis in angiosperms. Photosynth Res.

[63] Blomqvist LA, Ryberg M, Sundqvist C (2008). Proteomic analysis of highly purified prolamellar bodies reveals their significance in chloroplast development. Photosynth Res.

[64] Plöscher M, Reisinger V, Eichacker LA (2011). Proteomic comparison of etioplast and chloroplast protein complexes. J Proteome.

[65] Solymosi K, Andersson MX, Biswal B, Krupinska K, Biswal UC (2013). Etioplasts and their significance in chloroplast biogenesis. Plastid development in leaves during growth and senescence.

[66] Sakamoto W, Takami T (2018). Chloroplast DNA dynamics: copy number, quality control and degradation. Plant Cell Physiol.

[67] Finster S, Eggert E, Zoschke R, Weihe A, Schmitz-Linneweber C (2013). Light-dependent, plastome-wide association of the plastid-encoded RNA polymerase with chloroplast DNA. Plant J.

[68] Moon J, Zhu L, Shen H, Huq E (2008). PIF1 directly and indirectly regulates chlorophyll biosynthesis to optimize the greening process in Arabidopsis. Proc Natl Acad Sci.

[69] Zhong S, Shi T, Zhao Q, Zhao M, An F, Shi H (2009). EIN3/EIL1 cooperate with PIF1 to prevent photo-oxidation and to promote greening of Arabidopsis seedlings. Proc Natl Acad Sci.

[70] Szarejko I, Maluszynski M, Kasha KJ, Forster BP, Szarejko I (2003). Anther culture for doubled haploid production in barley (*Hordeum vulgare* L.). Doubled Haploid Production in Crop Plants.

[71] Li H, Devaux P (2005). Isolated microspore culture overperforms anther culture for green plant regeneration in barley (*Hordeum vulgare* L.). Acta Physiol Plant.

[72] Nakamura Y, Nakamura Y (2015). Biosynthesis of Reserve Starch.

[73] Yun MS, Kawagoe Y (2009). Amyloplast division progresses simultaneously at multiple sites in the endosperm of rice. Plant Cell Physiol.

[74] Du H, Tang D, Huang D (2013). Plastids division in shoot apical meristem during the tuberization of taro (*Colocasia esculenta*). Sci Hortic (Amsterdam).

[75] Enami K, Tanaka K, Hanaoka M (2012). Retrograde signals arise from reciprocal crosstalk within plastids. Plant Signal Behav.

[76] Rowan BA, Oldenburg DJ, Bendich AJ (2009). A multiple-method approach reveals a declining amount of chloroplast DNA during development in Arabidopsis. BMC Plant Biol.

[77] Kumar RA, Oldenburg DJ, Bendich AJ (2014). Changes in DNA damage, molecular integrity, and copy number for plastid DNA and mitochondrial DNA during maize development. J Exp Bot.

[78] Isono K, Niwa Y, Satoh K, Kobayashi H (1997). Evidence for transcriptional regulation of plastid photosynthesis genes in *Arabidopsis thaliana* roots. Plant Physiol.

[79] Maréchal A, Brisson N (2010). Recombination and the maintenance of plant organelle genome stability. New Phytol.

[80] Lepage E, Zampini E, Brisson N (2013). Plastid genome instability leads to reactive oxygen species production and plastid-to-nucleus retrograde signaling in Arabidopsis. Plant Physiol.

[81] Rowan BA, Oldenburg DJ, Bendich AJ (2010). RecA maintains the integrity of chloroplast DNA molecules in Arabidopsis. J Exp Bot.

[82] Cahoon AB, Harris FM, Stern DB (2004). Analysis of developing maize plastids reveals two mRNA stability classes correlating with RNA polymerase type. EMBO Rep.

[83] Legen J, Kemp S, Krause K, Profanter B, Herrmann RG, Maier RM (2002). Comparative analysis of plastid transcription profiles of entire plastid chromosomes from tobacco attributed to wild-type and PEP-deficient transcription machineries. Plant J.

[84] De Santis-Maciossek G, Kofer W, Bock A, Schoch S, Maier RM, Wanner G (1999). Targeted disruption of the plastid RNA polymerase genes *rpoA*, B and *C1*: molecular biology, biochemistry and ultrastructure. Plant J.

[85] Hricová A, Quesada V, Micol JL (2006). The *SCABRA3* nuclear gene encodes the plastid RpoTp RNA polymerase, which is required for chloroplast biogenesis and mesophyll cell proliferation in Arabidopsis. Plant Physiol.

[86] Lerbs-Mache S (2011). Function of plastid sigma factors in higher plants: regulation of gene expression or just preservation of constitutive transcription?. Plant Mol Biol.

[87] Xu YZ, Arrieta-Montiel MP, Virdi KS, de Paula WBM, Widhalm JR, Basset GJ (2011). Muts homolog1 is a nucleoid protein that alters mitochondrial and plastid properties and plant response to high light. Plant Cell.

[88] Maréchal A, Parent JS, Véronneau-Lafortune F, Joyeux A, Lang BF, Brisson N (2009). Whirly proteins maintain plastid genome stability in Arabidopsis. Proc Natl Acad Sci.

[89] Courtois F, Merendino L, Demarsy E, Mache R, Lerbs-Mache S (2007). Phage-type RNA polymerase RPOTmp transcribes the *rrn* operon from the PC promoter at early developmental stages in Arabidopsis. Plant Physiol.

[90] Sullivan J, Gray J (1999). Plastid translation is required for the expression of nuclear photosynthesis genes in the dark and in roots of the pea lip1 mutant. Plant Cell.

[91] Zubko M, Day A (2002). Differential regulation of genes transcribed by nucleus-encoded plastid RNA polymerase, and DNA amplification, within ribosome-deficient plastids in stable phenocopies of cereal albino mutants. Mol Gen Genomics.

[92] Emanuel C, Weihe A, Graner A, Hess W, Borner T (2004). Chloroplast development affects expression of phage-type RNA polymerases in barley leaves. Plant J.

[93] Hess WR, Prombona A, Fieder B, Subramanian AR, Börner T (1993). Chloroplast *rps15* and the *rpoB/C1/C2* gene cluster are strongly transcribed in ribosome-deficient plastids: evidence for a functioning non-chloroplast-encoded RNA polymerase. EMBO J.

[94] Hess WR, Hoch B, Zeltz P, Hübschmann T, Kössel H, Börner T (1994). Inefficient *rpl2* splicing in barley mutants with ribosome-deficient plastids. Plant Cell.

[95] Coronado MJ, Hensel G, Broeders S, Otto I, Kumlehn J (2005). Immature pollen-derived doubled haploid formation in barley cv. Golden Promise as a tool for transgene recombination. Acta Physiol Plant.

[96] Kumlehn J, Serazetdinova L, Hensel G, Becker D, Loerz H (2006). Genetic transformation of barley (*Hordeum vulgare* L.) via infection of androgenetic pollen cultures with *Agrobacterium tumefaciens*. Plant Biotechnol J.

[97] Untergasser A, Cutcutache I, Koressaar T, Ye J, Faircloth BC, Remm M (2012). Primer3-new capabilities and interfaces. Nucleic Acids Res.

[98] Ruijter JM, Ramakers C, Hoogaars WMH, Karlen Y, Bakker O, van den Hoff MJB (2009). Amplification efficiency: linking baseline and bias in the analysis of quantitative PCR data. Nucleic Acids Res.

[99] Livak KJ, Schmittgen TD (2001). Analysis of relative gene expression data using real-time quantitative PCR and the 2(−Delta Delta C(T)) method. Methods..

[100] Andersen CL, Jensen JL, Ørntoft TF (2006). Characterizing vascular parameters in hypoxic regions: a combined magnetic resonance and optical imaging study of a human prostate Cancer model. Cancer Res.

[101] Pfaffl MW, Tichopad A, Prgomet C, Neuvians TP (2004). Determination of stable housekeeping genes, differentially regulated target genes and sample integrity: BestKeeper - excel-based tool using pair-wise correlations. Biotechnol Lett.

[102] Doyle JJ, Doyle JL (1987). A rapid DNA isolation procedure for small quantities of fresh leaf tissue. Phytochem Bull.

