# Supplementary material for: Changes in plastid biogenesis leading to the formation of albino regenerants in barley microspore culture
Source: BMC Plant Biol. 2021 Jan 7;21:22. doi: 10.1186/s12870-020-02755-z (PMC7792217; doi:10.1186/s12870-020-02755-z)
Supplement: Supplementary file 1 — Additional file 1: Fig. S1. Expression profiles of genes related to translation occurring in plastid during isolated microspore culture of cvs. ‘Jersey’ and ‘Mercada’. Fig. S2. The relative expression profile of DPD1 gene encoding Mg2 + - dependent organelle exonuclease during microspore development of cvs. ‘Jersey’ and ‘Mercada’. Fig. S3. Plastid DNA content during regeneration of androgenic plants of cvs. ‘Jersey’ and ‘Mercada’. Fig. S4. The expression profiles of genes related to chloroplast differentiation during regeneration of plants of cvs. ‘Jersey’ and ‘Mercada’. Fig. S5. The plastids observed in converting embryos on 46dC of cvs. ‘Jersey’ and ‘Mercada’. Fig. S6. The relative expression level of genes related to plastid biogenesis, chloroplast differentiation and photosynthesis in albino regenerants of cv. ‘Mercada’ compared to albino regenerants of cv. ‘Jersey’. Fig. S7. The normalized expression level of genes important for plastid biogenesis during embryo formation and regeneration of androgenic plants. Table S1. List of genes and primers used to perform RT-qPCR analysis. Table S2. Composition of media used in isolated microspore culture. Table S3. List of genes, genome localisation and primers used to evaluate plastid DNA copy number using qPCR. [file 12870_2020_2755_MOESM1_ESM.pdf]

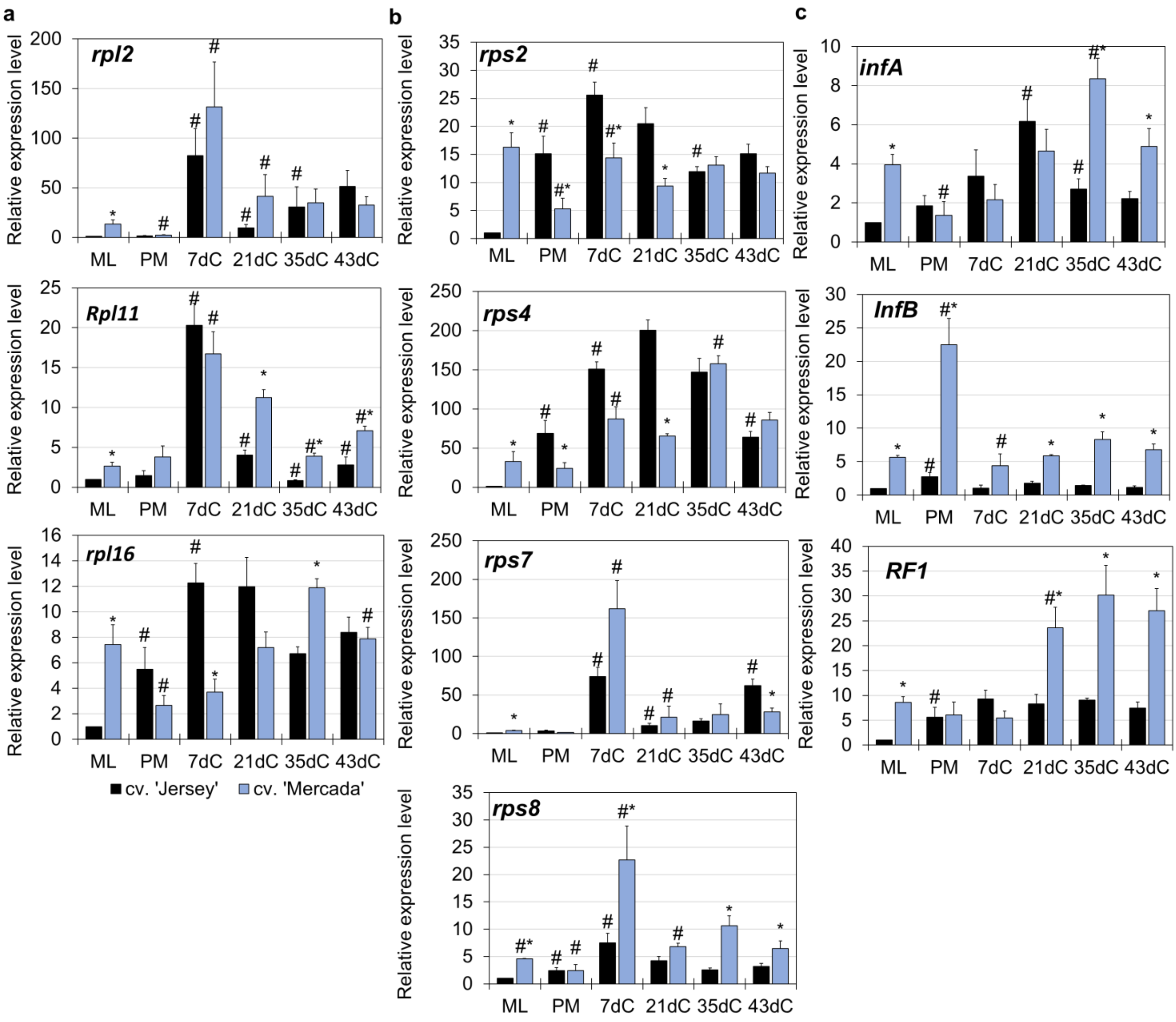

Figure S1. Expression profiles of genes related to translation occurring in plastid during isolated microspore culture of cvs. 'Jersey' and 'Mercada'.

a,b The relative expression level of genes encoding proteins of large (a) and small (b) ribosomes subunits. **c** Genes involved in initiation (*infA* and *InfB*) and termination (*RF1*) of translation. *Rpl11*, *InfB* and *RF1* are encoded in nuclear genome. Graphs show mean values of n ≥ 3 with SEM. Relative expression level normalised to ML microspores of cv. 'Jersey'. An asterisk presents a value significantly different between cultivars at a certain day of culture. A hash indicates a value significantly different from the preceding day of culture within cultivar (Tukey's test, $P < 0.05$). ML – mid-to-late microspore, PM – pre-treated microspores, dC – day of culture.

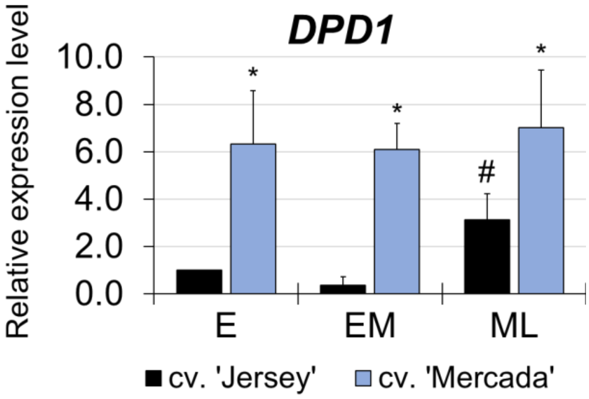

Figure S2. The relative expression profile of *DPD1* gene encoding Mg^{2+}-dependent organelle exonuclease during microspore development of cvs. ‘Jersey’ and ‘Mercada’.

Graph shows mean values of n ≥ 3 with SEM. Relative expression level normalised to E microspores of cv. ‘Jersey’. An asterisk presents a value significantly different between cultivars at a certain day of microspore development. A hash indicates a value significantly different from the preceding day of microspore development within cultivar (Tukey’s test, $P < 0.05$). Stages of pollen development: E – early, EM – early-mid, ML – mid-to-late.

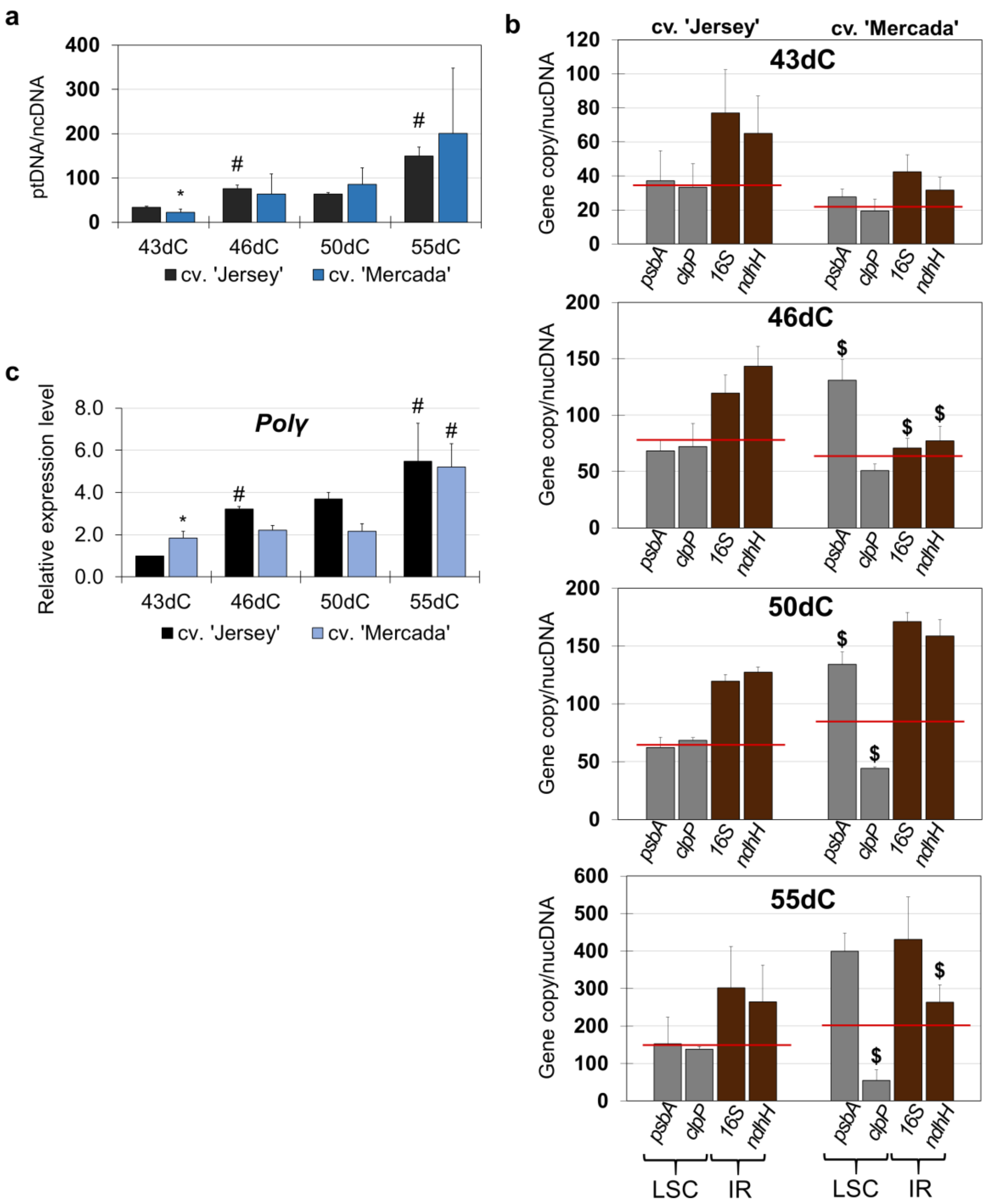

Figure S3. Plastid DNA content during regeneration of androgenic plants of cvs. 'Jersey' and 'Mercada'.

a Average plastome copy number in relation to nuclear genome in subsequent days of regenerating plants of 'Jersey' and 'Mercada' cultivars. **b** The individual copy number of genes localised in the plastid genome in subsequent days of isolated microspore culture of 'Jersey' and 'Mercada' cultivars in 'Jersey' and 'Mercada' cultivars. **c** The relative expression profile of *Polγ* (*Organellar DNA polymeraseI*) gene. Graphs show mean values of $n \geq 3$ with SD in a and b or SEM in c. Relative expression level normalised to 43dC of cv. 'Jersey'. An asterisk presents a value significantly different between cultivars at a certain day of culture. A hash indicates a value significantly different from the preceding day of culture within cultivar (Tukey's test, $P < 0.05$). A $ indicates value significantly different from the calculated average plastome copy number at a particular day of culture within cultivar (Student's t-test, $P < 0.05$). Red lines show the average copy numbers calculated from individual copies of presented genes. LSC – long single copy, IR – inverted repeat, SSC – short single copy, ML – mid-to-late microspore, PM – pre-treated microspores, dC – day of culture.

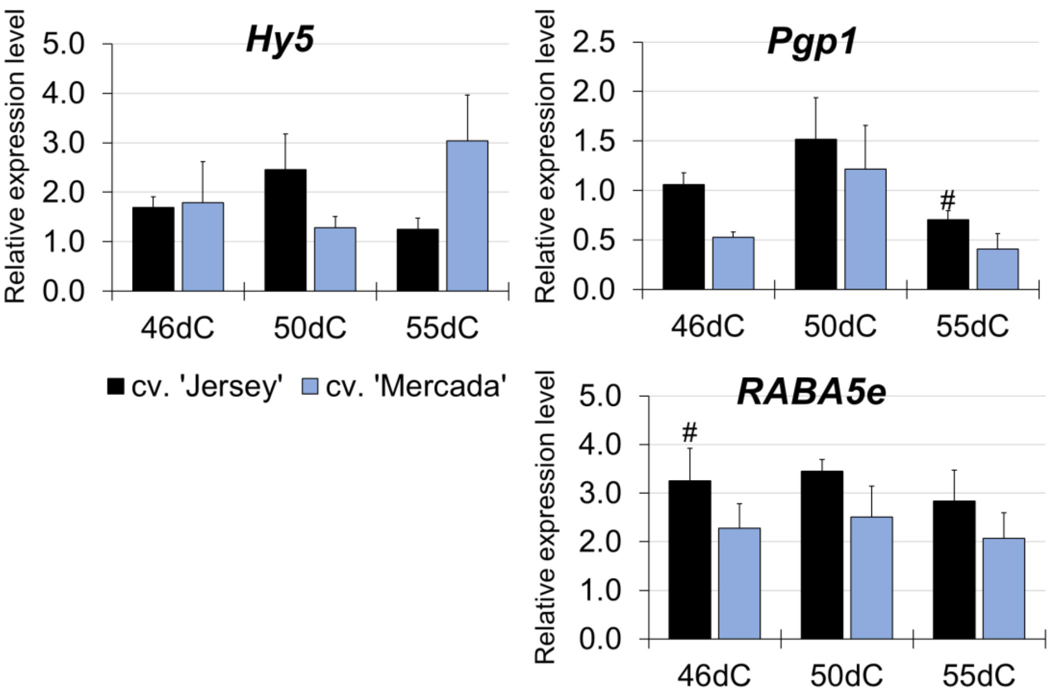

Figure S4. The expression profiles of genes related to chloroplast differentiation during regeneration of plants of cvs. 'Jersey' and 'Mercada'.
Gene encoding transcription factor HY5 and involved in synthesis (*PGP1*) and docking (*RABA5e*) of thylakoids in 'Jersey' and 'Mercada' cultivars. Graphs show mean values of n ≥ 3 with SEM. Relative expression level normalised to 43dC of cv. 'Jersey'. An asterisk presents a value significantly different between cultivars at a certain day of culture. A hash indicates a value significantly different from the preceding day of culture within cultivar (Tukey's test, $P < 0.05$). dC – day of culture.

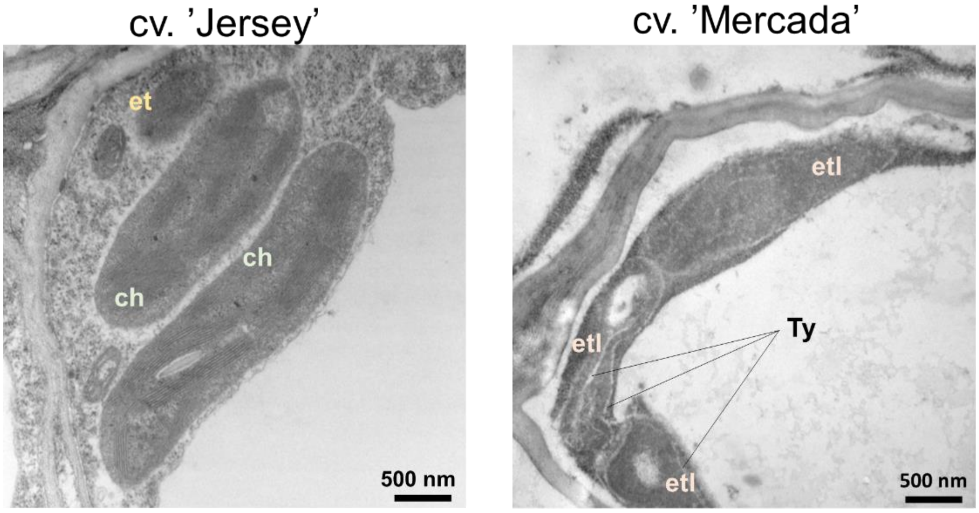

Figure S5. The plastids observed in converting embryos on 46dC of cvs. 'Jersey' and 'Mercada'. ch- chloroplast, et – etioplast, etl – etioplast-like plastid, Ty – thylakoid.

Chloroplasts that were observed only in cv. 'Jersey' are characterised by well-developed grana, whereas etioplast contain prolamellar body. Etioplast-like plastid in cv. 'Mercada' was more advanced in development and contained single perforated thylakoids and incipient grana without organized structure.

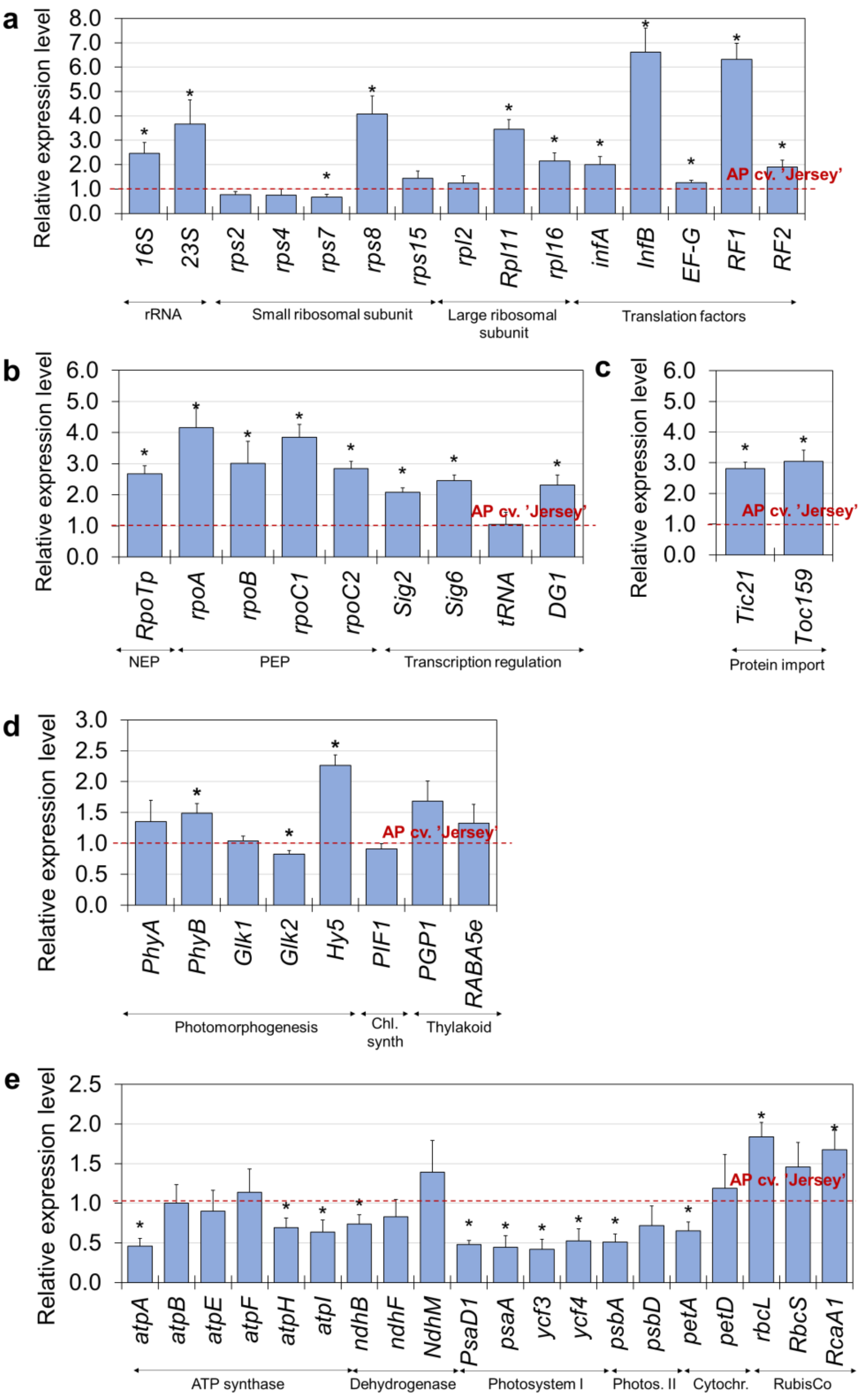

Figure S6. The relative expression level of genes related to plastid biogenesis, chloroplast differentiation and photosynthesis in albino regenerants of cv. 'Mercada' compared to albino regenerants of cv. 'Jersey'.

a-c The relative expression level of genes involved in transcription (a), protein import to plastid (b) and translation (c). **d** The relative expression level of genes related to chloroplast differentiation. **e** The relative expression level of genes related to photosynthesis. Graphs show mean values of $n \geq 3$ with SEM for relative expression level normalised to albino regenerants (GP) of cv. 'Jersey'. An asterisk presents a value significantly different between green regenerants of 'Jersey' and 'Mercada' cultivars (t-Student test, $P < 0.05$).

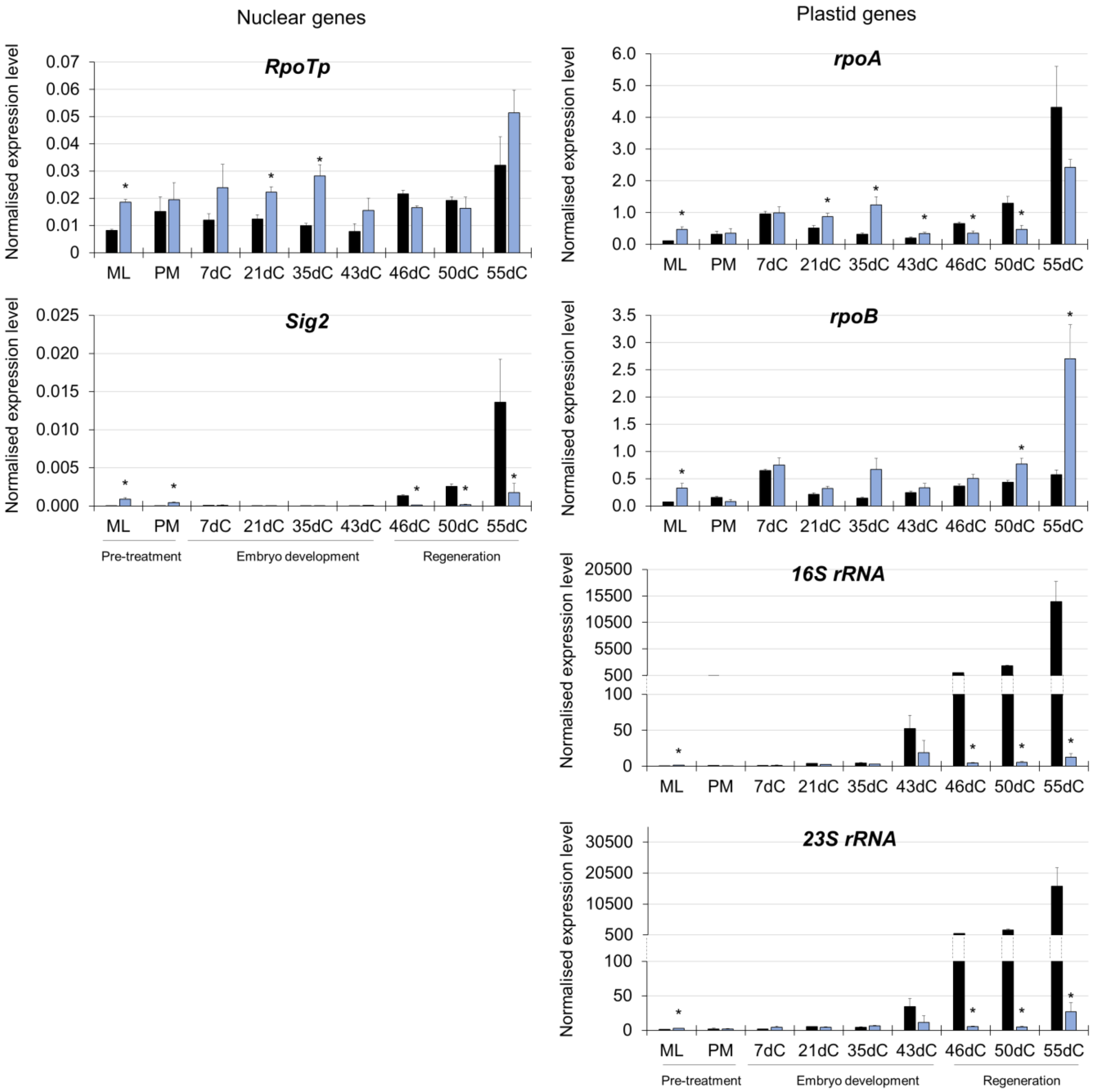

Figure S7. The normalized expression level of genes important for plastid biogenesis during embryo formation and regeneration of androgenic plants.

Graphs show mean values of n ≥ 3 with SEM for expression level normalised to reference genes. An asterisk presents a value significantly different between 'Jersey' and 'Mercada' cultivars (t-Student test, $P < 0.05$). ML – mid-to-late microspore, PM – pre-treated microspores, dC – day of culture.

Table S1. List of genes and primers used to perform RT-qPCR analysis

Process	Genome	Gene	Gene product	Gene ID or accession number	Primers 5'-3'		Ta [ºC]	Product [bp]
Transcription	Plastid	***rpoA***	RNA polymerase alpha subunit	4525081	pF	aagcgctttatgaggcttct	58	114
					pR	aaaggggtggagtaaacctg		
		rpoB	RNA polymerase beta subunit	4525093	pF	ggtcgacgaaatatgaatcg	58	101
					pR	ccaatcaaatgatccgtagc		
		rpoC1	RNA polymerase beta' subunit	4525121	pF	ttttgattagacgcatgcaa	58	95
					pR	gggaggaagaaccggtaata		
		rpoC2	RNA polymerase beta" subunit	4525122	pF	gcgaagaaataggttcgtca	58	86
					pR	tttcaatcgaaatcccaaaa		
		tRNA-Glu	tRNAE-UUC	4525087	pF	gcccctatcgtctagtggttc	58	69
					pR	cccaggggaagtcgaatc		
	Nuclear	***RpoTp***	RNA polymerase T phage-like	AJ507396.1	pF	tgccggaaacttgttaagag	58	114
					pR	tagcttcacttgggcttctg		
		Sig2	Sigma factor2	AK361731.1	pF	ttacatgcatgagaggctga	58	91
					pR	ctctgcgatgttttgtgttg		
		Sig6	Sigma factor6	AK251756.1	pF	aagatcgaagaaacgcaatg	58	92
					pR	ttggcctgtctttcttcttg		
Translation	Plastid	***23S rRNA***	23S rRNA	4525068	pF	gtcggcttgagtaacgaaaa	58	103
				4525179	pR	atcttaggccctgactcacc		
		16S rRNA	16S rRNA	4525063	pF	atgcattgagatcggaaaga	58	106
				4525101	pR	ctggggtctctaatcccatt		
		infA	Translation initiation factor1	4525109	pF	aacccgagagaagcaaaagt	58	103
					pR	agcctaggatgatggtgtca		
		rpl2	50S ribosomal protein L2	4525183	pF	ggtgctgtagcgaaactcat	58	115
				4525117	pR	tacccacttgtccgactgtt		
		rpl16	50S ribosomal protein L16	4525112	pF	ggtagccgttgttaaaccag	58	111
					pR	tgacttcgtatgggcatttt		
		rps2	30S ribosomal protein S2	4525123	pF	acgaattggtcgattacgaa	58	81
					pR	atggtggaattttcccattt		
		rps4	30S ribosomal protein S4	4525135	pF	ctggaaaagcaaaaaggtca	58	86
					pR	catacccaatcgaaaaagga		
		rps7	30S ribosomal protein S7	4525104	pF	cttgccattcgttggttatt	58	96
				4525061	pR	ccctttggcagcatctacta		
		rps8	30S ribosomal protein S8	4525110	pF	gacccggcttacgaatttat	58	112
					pR	cgagcttctcgatctgtcat		
	Nuclear	***cpEF-G***	Translation elongation factorG	AK353897.1	pF	ttgaggagttggctcaagac	58	108
					pR	ttgcctcatctggttcattt		
		InfB (cpIF2)	Translation initiation factor2	AK360218.1	pF	cagtagtggcagtggaaagg	58	86
					pR	ctttacgaagaacccgaaca		
		cpRF-1	Peptide release factor1	AK360613.1	pF	cagagaaggcatggaaagaa	58	109
					pR	acctctcccagattggaaac		
		Rpl11	50S ribosomal protein L11	AK369397.1	pF	tgacttgaactgcaaaacca	58	109
					pR	caccttctccttcttcacca		
Protein import to plastid	Nuclear	***Tic21***	Translocon at inner membrane of chloroplasts21	AK359467.1	pF	ctggcattctggtctcagtt	58	95
					pR	taaagggagacgttgcattc		
		Toc159	Translocase of chloroplast159	AK371279.1	pF	gggaggtgaagaggctaaag	58	96
					pR	actggactcgcatcactttc		
Replication	Nuclear	***Pol1***	Organellar DNA polymerase gamma	KM236205.1	pF	tgtaccagcacattcgtgac	57	127
					pR	gctttcctcctctcagaacc		
Plastid DNA degradation	Nuclear	***DPD1***	Defective in pollen organelle DNA degradation1	MLOC_11431.8	pF	acgccgtggtgatagaatta	58	118
					pR	accatgaacagctgcatttt		
Plastid division	Nuclear	***PDR1***	Plastid-dividing ring1	AK354363.1	pF	gatacctgggcttgtgaatg	57	99
					pR	tttttgctggaagaaacacc		
Reserve starch biosynthesis	Nuclear	***AgpS***	Glucose-1-phosphate adenylyltransferase small subunit	FN179369.1	pF	tcctggagctaatgacttcg	58	96
					pR	tcttcccagtaaccgtcgta		
		AgpL	Glucose-1-phosphate adenylyltransferase large subunit	X62242.1	pF	tcagacaagtgcaggatcaa	58	81
					pR	gatgatggagtgctcgattt		
		Dpe2	4-alpha-glucanotransferase2	FN179406.1	pF	tggcaggataattctgaagc	58	104
					pR	gtgagggaagaagactgcaa		
		GBSSI	Granule-bound starch synthaseI	AF486514.1	pF	cctgctacctcaagagcaac	58	111
					pR	caaagtcgtcgaaggagaag		
		Sbe1	Starch branching enzyme1	AY304541.1	pF	tagtttagggctgcgtgttc	58	94
					pR	gttttgcccaacatcatagc		
		SSII	Starch synthaseII	AY133249.1	pF	cattgacgctcctctcttc	58	87
					pR	agaacaaaatcatgcgcttc		
Assymilatory starch biosynthesis	Nuclear	***GBSSIb***	Granule-bound starch synthaseIb	AK368223.1	pF	atccaactgcaagggatgag	58	83
					pR	acaccctccctcactgtgtc		
		SSIIb	Starch synthaseIIb	FN179376.1	pF	ccaacttttgggttcacgag	58	102
					pR	tcggcaactccctatttgat		
		Glk1	Golden 2-like1	MLOC 43537	pF	gccgcgtctatgctctcatttg	58	69
					pR	agcaagagccaccaagattcgc		

Photomorph.	Nuclear	***Glk2***	Golden 2-like2	AK353571	pF	tggaattaccagaaggcaag	58	115
					pR	cccacgatgttgttcttctt		
		HY5	Long Hypocotyl5	HORVU6Hr1 G037760	pF	aaagcttatttgggcgatct	58	91
					pR	cattctgtagggtggaatgc		
		PhyA	Phytochrome photoreceptorsA	MLOC 81684	pF	gaggatggtctcatcactgg	58	107
					pR	cttcttagtgacgcctgctc		
		PhyB	Phytochrome photoreceptorsB	MLOC 9834	pF	tcgagaggagaagttgttgg	58	100
					pR	gggcaatcatgaactttgtc		
Chlorophyll synthesis	Nuclear	*PIF1*	Phytochrome-interacting factor1 (3-like5)	HV14044 2G00010	pF	gaggagacgagacaggatca	58	81
					pR	gacgccttgtcagtcttgtt		
Thylakoid synthesis and docing	Nuclear	*PGP1*	Phosphatidyl-glycerolphosphate synthetase1	MLOC 5438	pF	gagatttcctgctggatgtg	58	107
					pR	acgtcagtgtcgaagaagga		
	Nuclear	*RABA5e*	Rab GTPase protein5e	MLOC 73105	pF	ttcttcatggagacctcagc	58	101
					pR	ttcaggatcttcctgctcac		
Phosystem I	Plastid	*psbA*	PSII protein D1	4525096	pF	tttgggaagctgcatctgtt	58	110
					pR	ttcccactcacgacccatat		
		psbD	PSII protein D2	4525151	pF	gagttgccggagtattaggc	58	105
					pR	aagcacggaaggtatttgca		
Photosystem II	Plastid	*psaA*	PSI P700 apoprotein A1	4525132	pF	accgatattgcacaccatca	58	149
					pR	agacccttatggccttgtcc		
		ycf4	PSI asembly protein ycf4	4525190	pF	tcccttgactcgtactgatga	58	97
					pR	tacttcaattggtacgcgca		
		ycf3	PSI assembly protein ycf3	4525133	pF	cattaccgaggagaacaggc	57	113
					pR	taatttcccggagtaagcgc		
	Nuclear	*PsaD1*	PSI-D subunit of PSI	M98254.1	pF	aaggagcaggtcttcgagat	58	133
					pR	aggcgatcttgtacttggag		
NADH dehydrogenase	Plastid	*ndhB*	NADH-PQ oxidoreductase subunit2	4525105	pF	acgaatggcatcttcttctg	58	126
				4525060	pR	catatccgatttgccctatg		
		ndhF	NADH-PQ oxidoreductase subunit5	4525163	pF	tggggctaaaaacacttttg	58	137
					pR	ttccattatccattcctcca		
	Nuclear	*NdhM*	Subunit NDH-M of NAD(P)H:PQ dehydrogenase	AK357595.1	pF	tctatgacggggagatcaag	57	91
					pR	atctggctgctgttgaattt		
ATP synthase	Plastid	*atpA*	ATP synthase CF1 alpha subunit	4525127	pF	taatgggcgatgggttgatg	58	91
					pR	cccaagtaagcctcactcac		
		atpB	ATP synthase CF1 beta subunit	4525186	pF	cgggttgatgagaggaatgg	58	118
					pR	acccaaattgtcaacaggct		
		atpE	ATP synthase CF1 epsilon subunit	4525185	pF	aaccacgcccctattaacac	58	107
					pR	actattctcgcaaaaccgct		
		atpH	ATP synthase CF0 subunit III	4525125	pF	gctgcttctgttattgctgc	58	114
					pR	ttctggctgtctcgcaatac		
		atpI	ATP synthase CF0 subunit IV	4525124	pF	gaatccacaaaccatcccga	58	111
					pR	ggaacccagggaccatattc		
Cytochrome	Plastid	*petA*	Cytochrome f	4525192	pF	ttctttcccctgatcctgct	58	87
					pR	tctgtcctcttcctcggttt		
		petD	Cytochrome b_6/f complex subunit IV	4525080	pF	tgtaggtttagcggttctcg	58	107
					pR	tttgaaacacgggaaagaag		
RubisCo	Plastid	*rbcL*	RubisCo large subunit	4525187	pF	ctgcgggtacatgtgaagaa	58	90
					pR	ccccggttaagtagtcatgc		
	Nuclear	*RbcS*	RubisCo small subunit	AB020943.1	pF	agttcagcaaggttggcttc	58	100
					pR	catccgaacataggcagctt		
		RcaA1	RubisCo activase	M55446.1	pF	gatgtgctgcctcttcatca	57	81
					pR	tggttgttgacggtgtactg		
Reference genes		*ARF1*	ADP-ribosylation factor 1-like protein	AJ508228.2	pF	cgtgacgctgtgttgcttgt	58	61
					pR	ccgcattcatcgcattagg		
		EF1	Translation elongation factor 1-a	AJ472912	pF	ccctcctcttggtcgttttg	58	60
					pR	atgacaccaacagccacagttt		

Table S2. Composition of media used in isolated microspore culture

**Component (mg/L)**	**SMB1** (Starvation Medium Barley1)	**KBP** (Kumlehn's Barley Pollen)	**KBPD** (Kumlehn's Barley Pollen Differentiation)	**K4NB**
**Major elements**				
KNO_3	-	2020	2020	3640
NH_4NO_3	-	80	80	320
KH_2PO_4	-	340	340	340
$MgSO_4 \cdot 7H_2O$	-	246	246	246
$CaCl_2 \cdot 2H_2O$	147	441	441	441
**Trace elements**				
NaFeEDTA	-	27.5	27.5	20.6
$MnSO_4 \cdot H_2O$	8.4	8.4	8.4	8.4
H_3BO_3	3.1	3.1	3.1	3.1
$ZnSO_4 \cdot 7H_2O$	7.2	7.2	7.2	7.2
$CoCl_2 \cdot 6H_2O$	0.024	0.024	0.024	0.024
$CuSO_4 \cdot 5H_2O$	0.025	0.025	0.025	1.25
$Na_2MoO_4 \cdot 2H_2O$	0.12	0.12	0.12	0.12
KI	0.17	0.17	0.17	0.17
**Gamborg B5 vitamin mixture** (Duchefa Biochemie)				
Nicotinic acid	-	-	-	1
Pyridoxine-HCl	-	-	-	1
Thiamine-HCl	-	-	-	10
Myo-inositol	-	-	-	100
**Kao and Michayluk vitamin mixture** (Sigma)				
Retinol	-	0.01	0.01	-
Thiamine-HCl	-	1	1	-
Nicotinic acid	-	1	1	-
Riboflavin	-	0.2	0.2	-
Ca-pantothenate	-	1	1	-
Folic acid	-	0.4	0.4	-
Pyridoxine-HCl	-	1	1	-
Cobalamine	-	0.02	0.02	-
Ascorbic acid	-	2	2	-
Calciferol	-	0.01	0.01	-
Biotin	-	0.01	0.01	-
Choline chloride	-	1	1	-
p-Aminobenzoic acid	-	0.02	0.02	-
Myo-inositol	-	100	100	-
**Casein hydrolysate**	-	250	-	-
**Organic acids**				
Citric acid	-	40	-	-
Fumaric acid	-	40	-	-
Na-pyruvate	-	20	-	-
**NH_4Cl**	53.4	-	-	-
**Glutamine**	-	439.3	439.3	146.4
**BAP**	0.9	0.9	0.224	0.224
**Maltose**	144000	90000	90000	36000
**MES**	424.4	-	-	-
**Phytagel**	-	-	8000	6000
**Cefotaxime**	250	250	-	-
**pH**	5.5	5.9	5.9	5.8

Table S3. List of genes, genome localisation and primers used to evaluate plastid DNA copy number using qPCR

(a) Plastid genes							
**Gene**	**Encoded factor**	**Plastome localisation**	**Region**	**Gene ID**	**Primers 5'-3'**		**Product [bp]**
psbA	Photosystem II protein D1	619..1680, cmpl	LSC	4525096	pF	tttgggaagctgcatctgtt	110
					pR	ttcccactcacgacccatat	
matK	Maturase K	2206..3741 cmpl	LSC	4525145	pF	cgataccatagtccccgcta	88
					pR	gggtttactaataggatgcccc	
psbD	Photosystem II protein D2	9159..10220	LSC	4525151	pF	gagttgccggagtattaggc	105
					pR	aagcacggaaggtatttgca	
atpI	ATP synthase CF0 subunit IV	31348..32091	LSC	4525124	pF	gaatccacaaaccatcccga	111
					pR	ggaacccagggaccatattc	
clpP	ATP-dependent Clp protease proteolytic subunit	68250..68900, cmpl	LSC	4525074	pF	cctggagatgaagaagcgac	88
					pR	cgcaacgaatctcttgacct	
infA	Translation initiation factor1	76802..77143 cmpl	LSC	4525109	pF	aacccgagagaagcaaaagt	103
					pR	agcctaggatgatggtgtca	
*ndhB**	NADH-plastoquinone oxidoreductase subunit2	86790..89034, cmpl	IR	4525060	pF	agtgtggttcgttcgacaaa	105
		129100..131344		4525105	pR	cggagtggggatagcatttc	
*16S rRNA**	16S rRNA	92685..94176	IR	4525063	pF	atgcattgagatcggaaaga	106
		123958..125449 cmpl			pR	ctggggtctctaatcccatt	
*ndhH**	NADH-plastoquinone oxidoreductase subunit7	102501..102707	IR	4525162	pF	tagtcaatatgggccctcaa	82
		114452..115633, cmpl		4525173	pR	tcaataacatcctcaccatcaa	
ndhF	NADH-plastoquinone oxidoreductase subunit5	102776..104995, cmpl	SSC	4525163	pF	tggggctaaaaacacttttg	137
					pR	ttccattatccattcctcca	

(b) Nuclear genes							
**Gene**	**Encoded factor**	**Number of copy in nuclear genome**	**Accession number**	**Primers 5'-3'**		**Product [bp]**	**Reference**
ARF1	ADP-ribosylation factor 1-like protein	1	AJ508228.2	pF	cgtgacgctgtgttgcttgt	61	Rapacz *et al*., 2012
				pR	ccgcattcatcgcattagg		
EF1	Translation elongation factor 1-a	1	AJ472912	pF	ccctcctcttggtcgttttg	60	
				pR	atgacaccaacagccacagttt		

LSC – long single copy, IR – inverted repeat, SSC – short single copy.
